# Can self-referential information improve directed forgetting? Evidence from a multinomial processing tree model

**DOI:** 10.1371/journal.pone.0211280

**Published:** 2019-01-28

**Authors:** Runzhou Wang, Yaowu Song, Xiaojun Zhao

**Affiliations:** School of Education, Hebei University, Baoding, China; Indiana University Bloomington, UNITED STATES

## Abstract

A large body of research has shown that self-referential processing can enhance an individual’s memory of information. However, there are many arguments about how self-referential processing affects directed forgetting (DF). In this study, two experiments were designed to investigate the DF effect and its internal psychological mechanism under explicit and implicit referential conditions using the item-method DF paradigm combined with the storage-retrieval MPT model. We compare the difference in the DF effect between self-referential and other-referential conditions and explain the reasons for the difference. Our results suggest that the item-method DF effect is the result of a selective rehearsal mechanism and a retrieval inhibition mechanism working together. Both self-reference and other-reference can cause DF in explicit referential processing or implicit referential processing, although the DF effect is stronger under the self-referential condition. Furthermore, the memory advantage effect of implicit self-referential processing is stronger than that of explicit self-referential processing.

## Introduction

As an aspect of memory, forgetting plays a crucial role in the process of cognitive processing. In daily life, individuals must adjust their memory according to changes in external environmental information. For example, when performing a programmatic task, one may misremember the operation of a key step. If one finds that this information is wrong, he or she must forget it in order to prevent erroneous information from occupying limited cognitive resources, resulting in memory overload [1]. This emphasis on directional and intentional forgetting is called directed forgetting (DF), which means that forgotten instructions cause memory impairment [2]. Previous studies have found that intentionally forgotten interference information can help individuals perform current tasks more efficiently, while proactively forgetting self-related negative emotions and traumatic events has a positive effect on mental health [3, 4].

### Can self-referential information lead to directed forgetting?

Rogers, Kuiper, and Kirker (1977) demonstrated that when the content of learning materials is associated with the self, memory performance is better than it is in other encoding conditions [5]. Participants are more likely to remember the birthday of a friend if the friend’s birthday is close to their own, and they are more likely to forget the friend’s birthday if it is distant [6]. However, Gutchess, Kensinger, and Schacter (2010) found that young and older adults both engage a similar network of regions during self-referential processing, but young exhibit subsequent forgetting effects in prefrontal and parietal regions [7]. Can self-referential information induce DF?

Researchers hold two different views on how self-referential information affects intentional forgetting. One view focuses on explaining the effect of self-referential processing on DF from the perspective of encoding. It is believed that self-referential effects promote the recall of TBF (to-be-forgotten) items under a self-reference condition by enhancing the encoding of TBF items [8], which suggests that self-referential information impairs DF effects [9]. Liang (2008) found that participants could not directly forget emotional adjectives under a self-reference condition [10]. Specifically, participants’ encoding of learning materials was more meticulous under the self-referential condition and less inhibited during the testing phase. Thus, DF effects were impaired. Yang et al. (2013) used the list-method DF paradigm and the fMRI technique to examine the relationship between the DF effect of negative trait adjectives under a self-referential condition and an other-referential condition. The authors observed the DF phenomenon under both reference conditions, although the DF effect was smaller under the self-referential condition than under the other-referential condition. This is because self-referential information is more difficult to forget, and its rate of recognition is higher. Similarly, Mao et al. (2017) used the list-method DF paradigm and ERP technology to explore the retrieval inhibition mechanism of DF of self-referential information. They found that self-referential processing enhanced the familiarity of learning materials and alleviated retrieval inhibition, which supported the idea that self-referential processing impairs DF effects. Additionally, in a study of retrieval-induced forgetting, researchers found that learning materials under a self-reference condition did not cause retrieval-induced forgetting, but an other-referential condition did. These studies showed that under a self-referential condition, learning materials are processed with finer encoding, and the retrieval practice cannot inhibit these materials [11, 12]. The diminished DF effect in the self-referential condition in these studies can be explained by participants’ greater activity in encoding self-related information, including the use of elaborate and organized memory strategies and improved subsequent retrieval of memory content [7, 13]. These strategies also reduced the inhibition effect of information retrieval.

The other view focuses on explaining the effect of self-referential processing on DF from the perspective of inhibition and concludes that DF effects under self-referential conditions will be enhanced. People with good memory can choose the information that they need to remember and can ignore or inhibit the information that they need to forget [14]. Most studies supporting this view have found that participants showed DF under a self-referential condition but not under an other-reference condition [14–17]. The enhancement of DF effects under a self-referential condition may suggest that individuals more elaborately encode information that is associated with the self at the level of consciousness. In the course of the experiment, participants used means to distinguish between TBF items and TBR (to-be-remembered) items. Self-related TBF items and TBR items had higher discrimination due to the elaborate encoding. Therefore, the TBR items used more cognitive resources and rehearsal opportunities, while the TBF items were forgotten by the instruction-induced inhibition mechanism and were excluded from the memory system, causing DF [14]. However, under an other-reference condition, learning materials cannot be uniquely processed as in a self-reference condition. Therefore, discrimination between learning materials is low, which leads to a non-significant effect of DF [17].

Therefore, it remains unclear whether self-reference and other-reference cause DF and whether differences in the DF effect under these two conditions can be explained by different internal psychological mechanisms. Data from traditional behavioral experiments can only reflect the amount of DF costs (In the list-method paradigm, participants who were instructed to forget List 1 recall fewer items from that list compare with the remember group) and DF benefits (In the list-method paradigm, participants who were instructed to forget List 1 recall more items from List 2 compare with the remember group), and electrophysiological and neuroimaging studies can only illustrate the cerebral functional areas that are activated. Memory is a dynamic mental process that includes encoding, storage, and retrieval. Forgetting as an aspect of memory can also be explained by this process. The controversy about the DF effect under a self-referential condition focuses on the encoding and retrieval of learning materials. Thus, it is important to quantify storage and retrieval in the process of memory and to compare storage quantity and extraction quantity under different referential conditions. In this way, we can account for the causes and underlying psychological mechanisms of DF. According to these different perspectives, we propose two opposing hypotheses:

H1. Self-referential information impairs the DF effect, supporting the perspective of encoding.

H2. Self-referential information enhances the DF effect, supporting the perspective of inhibition.

In the present study, to ensure that the experimental situation closely reflected people’s lives and to improve the ecological validity, we first chose the item-method DF paradigm as the experimental paradigm. Life events enter the cognitive processing system sequentially, and individuals must determine whether each event needs to be remembered or forgotten. For example, a man orders a medium-well steak and a salad. After brief consideration, he decides to change the medium-well steak to a medium steak. At the same time, the guests at the next table ask the waiter for a set of tableware. Therefore, the waiter must first remember the “medium-well steak” and the “salad” and then forget the “medium-well steak,” remember the “medium steak,” and finally remember that the “guests need a set of tableware.” This example is similar to the item-method paradigm. In contrast, the psychological mechanisms of the list-method paradigm may also involve context change and require participants to remember or forget large amounts of information simultaneously; therefore, there is no item-method paradigm that reflects the reality of life. However, the psychological mechanism of item-method DF is controversial. Researchers who support selective rehearsal accounts find that delays in the presentation of instructions do not affect DF effects. It was found that participants conducted maintenance rehearsal on learning materials without deep encoding before instructions were presented, and they selectively rehearsed TBR items after instructions were presented [18]. Zack, Radvansky, and Hasher (1996) found deteriorating inhibitory control abilities in the elderly, for whom inhibiting the retrieval of TBF items was difficult [19]. Thus, the DF effects were weaker for elderly than for young people, providing support for the retrieval inhibition account. However, many researchers believe that DF effects are the result of these two types of mechanisms working together [20–23]. Thus, we propose the following hypothesis:

H3. DF effects are the result of selective rehearsal and retrieval inhibition.

Most previous studies have used emotional adjectives and trait adjectives as materials [17]. However, information in daily life rarely involves positive or negative personality traits, and positive or negative emotions and personality traits can affect research results. The present study does not involve a discussion of emotions and personality traits. Because Mao et al. (2017) found that participants also experienced DF of specific nouns under a self-referential condition [8, 24], we chose specific nouns that were related closely to daily life as materials.

### A quantitative study of the mechanism of directed forgetting using multinomial models

Previous research has produced controversy over the DF effect because there is no suitable method to quantitatively study the internal psychological mechanism of DF. Rummel et al. (2016) applied multinomial models to quantify storage and retrieval in the DF process [20]. This provided the basis for our study of the psychological mechanism of DF. Thus, multinomial models can be used to explain the DF phenomenon under self-referential conditions.

**Fig 1:**
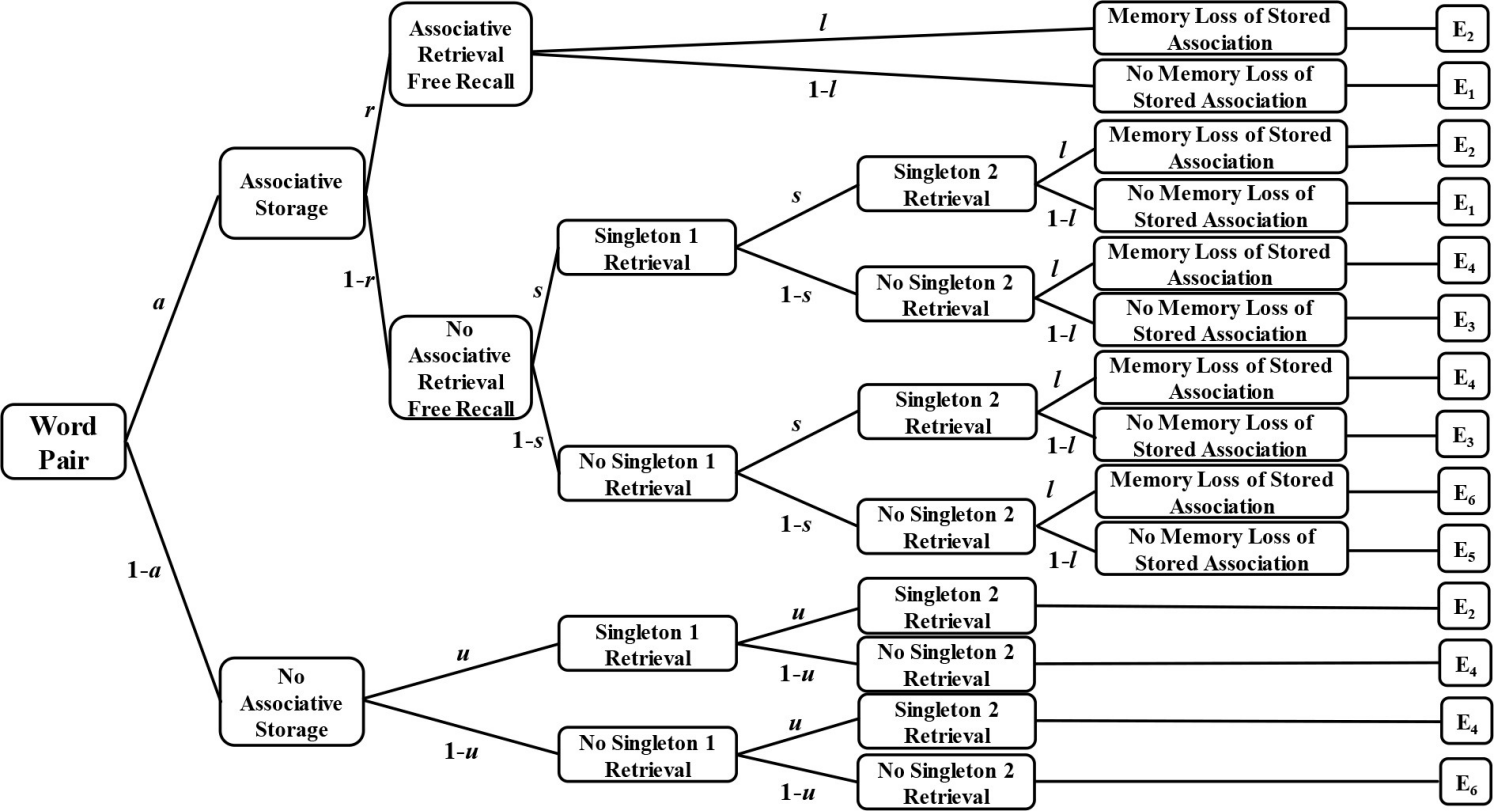
Storage-retrieval MPT model.

Multinomial processing tree (MPT) models are a class of mathematical models for manipulating categorical data so that potential cognitive processes can be separated from observed behavioral phenomena [25]. The MPT model we used in the present study is the storage-retrieval MPT model (Fig 1), which is often combined with the free-then-cued-recall paradigm to study memory of word pairs [26, 27]. The experimental procedure of this paradigm is as follows: learning the word pairs, performing a distraction task, performing a free-recall test, and finally taking the first word of each word pair as a cue to perform a cue-recall test. The test revealed one of the following six types of behavioral events for each word pair: both items in the word pair are free recalled with successful cued recall (E_1_); both words in the word pair are free recalled with failed cued recall (E_2_); a single item in the word pair is free recalled with successful cued recall (E_3_); a single item in the word pair is free recalled with failed cued recall (E_4_); no item in the word pair is free recalled with successful cued recall (E_5_); and no item in the word pair is free recalled with successful cued recall (E_6_). E_1_~E_6_ reflect the five potential cognitive statuses of the participants, which are represented by parameters a, r, s, u, and l, respectively. The range of each parameter is [0,1]. Storage parameter a indicates the probability of storage of two items in a word pair, including remembering two items and their association and maintaining the memory until the test is completed. Retrieval parameter r indicates the probability of the retrieval of two items that were stored associatively in a word pair during the free-recall test. Parameters s, u, and l are used as additional parameters to eliminate unrelated interference factors in order to increase model validity but not differentiate storage and retrieval processes. Parameter s indicates the probability that two items of a word pair are retrieved as singletons in the free-recall test even though they were stored as a pair. Parameter u reflects the probability that both items of a word pair are retrieved as singletons in the free-recall test without being stored associatively. Parameter l indicates that some items are lost from memory during the delay between the free-recall and the cued-recall test. Parameters s and u reflect that the cognitive states in the standard free-then-cued-recall paradigm occur rather infrequently so that values are small, and parameter estimates for l are usually close to zero [20].

The model in Fig 1 can be transformed into the following six standardized equations that express the probabilities for each recall event as a combination of the underlying cognitive states:

$$P(E_{1})=ar(1-l)+a(1-r)s^{2}(1-l)$$ (1)

$$P(E_{2})=arl+a(1-r)s^{2}l+(1-a)u^{2}$$ (2)

$$P(E_{3})=2a(1-r)s(1-s)(1-l)$$ (3)

$$P(E_{4})=2a(1-r)s(1-s)l+2(1-a)u(1-u)$$ (4)

$$P(E_{5})=a(1-r){\left(1-s\right)}^{2}(1-l)$$ (5)

$$P(E_{6})=a(1-r){\left(1-s\right)}^{2}l+(1-a){\left(1-u\right)}^{2}$$ (6)

## Experiment 1

The purpose of Experiment 1 is to examine whether DF will occur and what the internal psychological mechanism of DF is under explicit self-reference or other-reference conditions. In addition, we compare the differences between the DF effects of these two referential conditions and consider what type of mechanism causes the differences. Finally, we provide quantitative explanations.

### Method

#### Ethics statement

The study was approved by the local ethics committee of Hebei University in China. All participants provided verbal informed consent prior to their inclusion in the study.

#### Participants

Thirty-four students from a university in Hebei province (19 women; M_age_ = 21.56 years, SD_age_ = 3.26, range: 18–30 years) participated in this experiment. All participants had normal intelligence and normal or corrected-to-normal vision and reported no current or past neurological or psychiatric disease. Each participant received monetary compensation and small gifts after the experiment. Because the item-method paradigm does not allow researchers to distinguish between DF costs and DF benefits as the list-method paradigm does, researchers have proposed adding a control group that receives TBR cues for all items to the item-method paradigm [20, 28–32]. According to this method, we divided the participants into two groups (experimental group and control group) with n = 17 participants each.

#### Materials

According to the interrelationships between things (e.g., sky—wild goose; sky—seagull), we selected the two-character Chinese nouns of medium frequency [33] from the Modem Chinese Frequency Dictionary [34]. The word frequency of all nouns was controlled between 3×10^−4^ and 9.89×10^−3^.

First, we selected 48 nouns as cue words. Forty-eight nouns that matched the cue words in a relationship were found as target words to form 48 cue-target word pairs (controlling the initials, finals, number of strokes of cue words, and target words). To control material-specific effects, we found another 48 nouns that matched the cue words as new target words instead of the original target words (see Rummel et al., 2016, for a similar method). Whether the new target words were used was counterbalanced. In addition, we selected four word pairs for the practice phase, for a total of 152 two-character Chinese nouns in Experiment 1. In the actual learning phase, the participants in the control group were instructed to remember all word pairs. The experimental group was asked to remember one half of the word pairs and to forget the other half (two types of instructions sets were counterbalanced within the experimental group). The pairs of words that were used for the self-reference and other-reference were equal to each other (referential category sets were counterbalanced within each group). The average word frequency of word pairs and the total number of strokes of word pairs were submitted to a 2 (instruction: TBF vs. TBR) × 2 (reference: self-reference vs. other-reference) two-factor completely random design analysis of variance (ANOVA). The analysis showed that the total number of strokes of word pairs consisting of cue words and original target words had no main effects and interactions of instruction or reference (all ps > 0.167). This was the same as the average word frequency (all ps > 0.148). The analysis showed that the total number of strokes of word pairs consisting of cue words and new target words had no main effects and interactions of instruction or reference (all ps > 0.259). This was the same as the average word frequency (all ps > 0.399). To induce the self-reference or other-reference of the participants, we presented a sentence to the participants before the appearance of cue-target word pairs. This sentence described a state of the participants themselves or that of a stranger named Li Hua (e.g., “I put the cup in the kitchen” or “Li Hua went to the bookstore to buy a magazine”). In each sentence, we marked the personal pronouns in red to highlight the referential condition. The cue word and target word were also marked red. In the experimental group, the TBF pairs were called TBF/R items, and the TBR pairs were called TBR/R items. In the control group, all word pairs were TBR pairs. The word pairs corresponding to the TBF pairs of the experimental group were called TBF/R items, and the word pairs corresponding to the TBR pairs of the experimental group were called TBR/R items (corresponding method sets were counterbalanced within each control group).

#### Design

The design was a 2 (group: experimental group vs. control group) × 2 (reference: self-reference vs. other-reference) × 2 (item type: TBF/R vs. TBR/R) mixed design with a within-subjects factor presentation reference and item type and a between-subjects factor group.

#### Procedure

**Fig 2:**
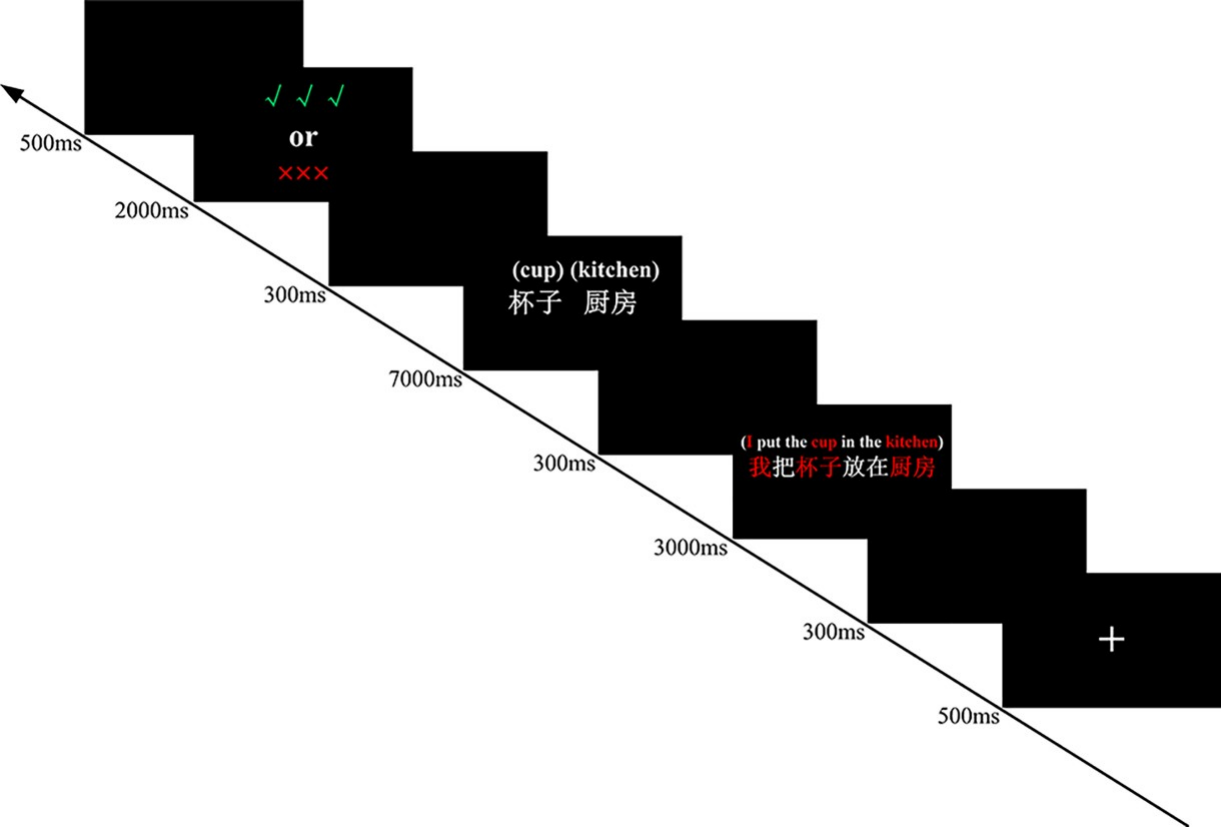
The procedure of Experiment 1.

The experimental programs were written and run using E-prime 2.0, and the materials were presented on a Dell 23.8-inch LED monitor with a screen resolution of 1920 × 1080 pixels. The word pairs and sentences used SimSun 36-pt font with a black background. The experiment was divided into four phases. The first phase was the practice phase. Its purpose was to allow participants to quickly understand the experimental process. Before the beginning of the practice, experimenters read the instructions to inform the participants that it was a test of memory and then explained the experimental procedure: “First, a fixation point will appear in the center of the screen, and then a sentence that may describe yourself or a stranger (in this experiment, we call him ‘Li Hua’) will be presented in the same place. You just need to read this sentence silently and imagine yourself or a stranger ‘Li Hua’ at the state described in that sentence. This is extremely important to our experiment. Then, a white word pair will be presented on the left and right sides of the center of the screen, which are the red-colored nouns in the previous sentence except for the personal pronouns. You need to learn the word pair. There will be an instruction after a word pair appears for a period. If it is red ‘×××,’ please forget that word pair. If it is green ‘√√√,’ please remember that word pair. Throughout the experiment, please try your best to memorize the word pairs you need to remember to complete the subsequent test.” After a brief practice, participants were required to verbally report the TBR pairs. The second phase was the actual learning phase. Before the learning began, the participants were told that the procedure of the actual learning phase was the same as that of the practice phase, except that there was a greater number of word pairs that needed to be memorized. Moreover, participants needed to forget the word pairs they had memorized during the practice phase and attempt to memorize the following word pairs. At the beginning of each trial, there was a 500 ms fixation point, after which a sentence was presented for a duration of 3000 ms. Next, a word pair was displayed for 7000 ms, and an instruction (√√√ or ×××) followed by the word pair was displayed for 2000 ms. To avoid fatigue caused by the continuous visual stimulus, we added a 300 ms blank interval between each visual stimulus, with an interval of 500 ms between each trial (see Fig 2). In the experimental group, 24 word pairs were used for self-reference, and 24 word pairs were used for other-reference. Half of participants in each of the two referential conditions followed instructions to forget. The control group used the same settings as the experimental group except that all word pairs followed the instruction to remember. After all materials were presented in the actual learning phase, participants completed a 60-s double-digit addition and subtraction task on answer sheet 1 to control the influence of the recency effect. The final phase was the test phase, and participants were asked to recall as many word pairs as they could remember during the 180-s free-recall test on answer sheet 2, regardless of the memory instruction (√√√ vs. ×××) and in any order. Word pairs could be recalled in pairs; if the participants remembered only singleton words in a word pair, these could also be recalled as singleton words. At the end of the free-recall test, the participants completed a 180-s cue recall test in which the first word of each word pair from the actual learning phase was presented (randomly intermixed) on answer sheet 3, and the participants needed to write the second word.

### Results and discussion

#### Behavioral data analyses

**Fig 3:**
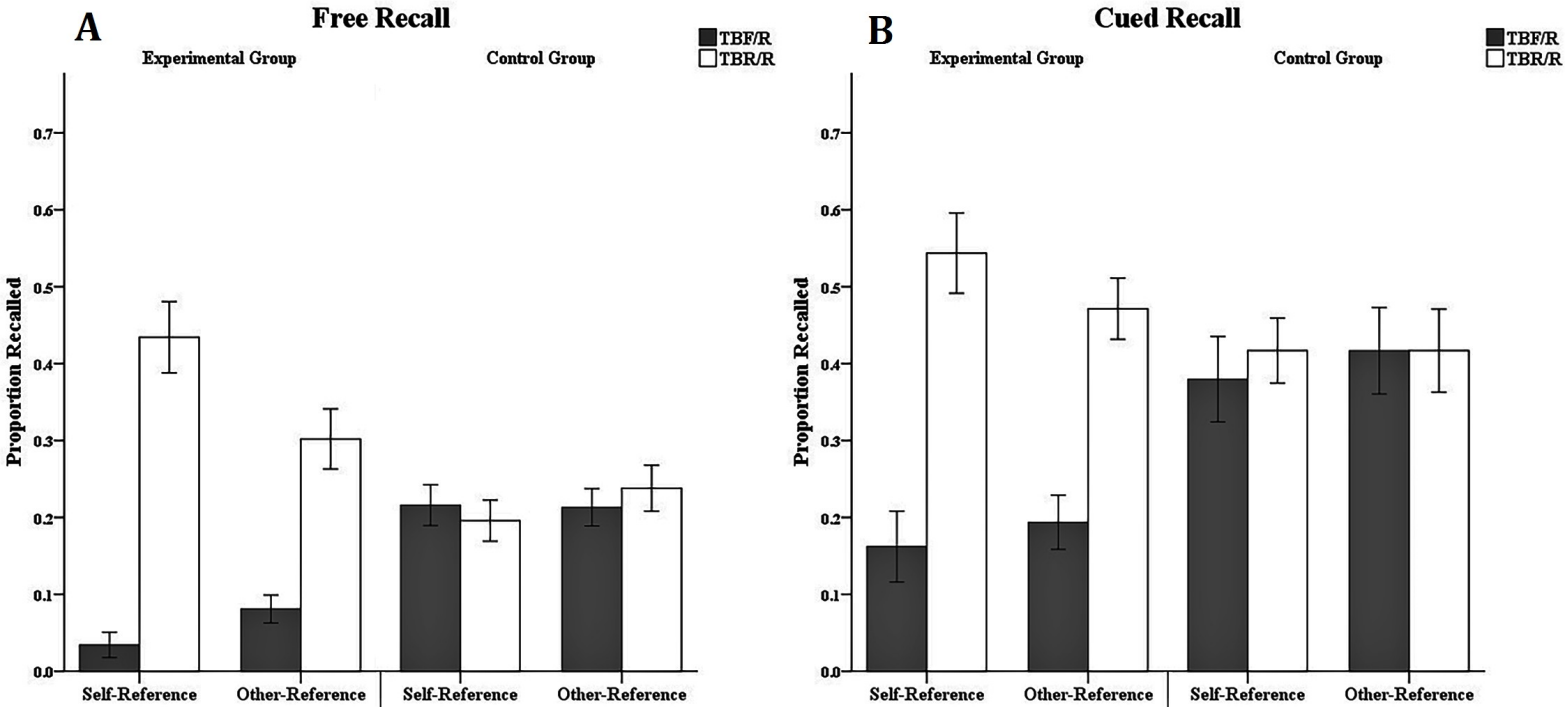
Mean proportion of TBF/R-item and TBR/R-item recall in Experiment 1. Mean proportion of TBF/R-item and TBR/R-item recall as a function of referential categories (self-reference, other-reference), group categories (experimental group, control group) and memory instruction (forget, remember) for free recall (Fig 3A) and cued recall (Fig 3B) in Experiment 1. Error bars depict standard errors.

Fig 3 shows the results of the ANOVA with the dependent variables of the free-recall rate of the word pairs and the cue-recall rate of target words under different referential conditions.

**Free-recall performance:** Free-recall rates were submitted to a 2 (group: experimental group vs. control group) × 2 (reference: self-reference vs. other-reference) × 2 (item type: TBF/R vs. TBR/R) mixed-factorial ANOVA with a within-subjects factor presentation reference and item type and a between-subjects factor group. The analysis indicated no main effect of group, F (1, 32) = 0.01, p > 0.05, and no difference in free-recall rates between the experimental group (M = 0.21, SE = 0.02) and the control group (M = 0.22, SE = 0.02). The analysis indicated no main effect of reference, F (1, 32) = 2.22, p > 0.05, and no difference in free-recall rates between the self-referential materials (M = 0.22, SE = 0.02) and other-referential materials (M = 0.21, SE = 0.02). However, there was a significant main effect of item type, F (1, 32) = 48.76, p < 0.001, η_p_^2^ = 0.60. The free-recall rate of TBF/R items (M = 0.14, SE = 0.01) was significantly lower than that of TBR/R items (M = 0.29, SE = 0.02). The interaction between group and reference was not significant, F (1, 32) = 2.22, p > 0.05. There was a significant interaction between group and item type, F (1, 32) = 42.28, p < 0.001, η_p_^2^ = 0.60. The simple effect analysis showed that the free-recall rate of TBF/R items (M = 0.06, SE = 0.02) was significantly lower than that of the TBR/R items (M = 0.37, SE = 0.03), p < 0.001, η_p_^2^ = 0.75, in the experimental group, but there was no difference in free-recall rates between TBF/R items (M = 0.22, SE = 0.02) and TBR/R items (M = 0.22, SE = 0.03) in the control group, p > 0.05. There was a significant interaction between reference and item type, F (1, 32) = 5.97, p = 0.020, η_p_^2^ = 0.16. The simple effect analysis showed that the free-recall rate of TBF/R items (M = 0.13, SE = 0.02) in the self-referential conditions was significantly lower than that of the TBR/R items (M = 0.32, SE = 0.03), p < 0.001, η_p_^2^ = 0.57, and the free-recall rate of TBF/R items (M = 0.15, SE = 0.02) in the other-referential conditions was significantly lower than that of the TBR/R items (M = 0.27, SE = 0.03), p < 0.001, η_p_^2^ = 0.48. The three-way interaction effect between group, reference, and item type was significant, F (1, 32) = 16.66, p < 0.001, η_p_^2^ = 0.34. We conducted pairwise comparisons for the analysis of the free-recall rates and found that the free-recall rate of TBF/R items (M = 0.03, SE = 0.02) was significantly lower than that of the TBR/R items (M = 0.43, SE = 0.04), p < 0.001, η_p_^2^ = 0.74, in the experimental group under the self-referential condition. Additionally, the free-recall rate of TBF/R items (M = 0.08, SE = 0.02) was significantly lower than that of TBR/R items under the other-referential conditions (M = 0.30, SE = 0.04), p < 0.001, η_p_^2^ = 0.59. There was no significant difference between the free-recall rates of TBF/R items and TBR/R items in the control group in either the self-referential or the other-referential condition, Fs < 1. We used pairwise comparisons to compare the costs and benefits of DF under different referential conditions. The results showed that there were significant DF costs [F (1, 32) = 34.02, p < 0.001, η_p_^2^ = 0.52] and DF benefits [F (1, 32) = 19.94, p < 0.001, η_p_^2^ = 0.38] under the self-referential condition. Under the other-reference condition, DF costs were significant [F (1, 32) = 19.01, p < 0.001, η_p_^2^ = 0.37], but DF benefits were not significant [F (1, 32) = 1.68, p > 0.05].

To further analyze the data, we conducted an independent-sample t-test with the DF effect under the self-referential condition and the other-referential condition in the experimental group. The result showed that the DF effect (M = 0.40, SE = 0.05) under the self-referential condition was stronger than the DF effect (M = 0.22, SE = 0.04) under the other-referential condition, t (32) = 2.86, p = 0.007, d = 0.98. The independent-sample t-test revealed no significant difference between the DF costs under the self-referential condition and the other-referential condition, t (32) = 1.17, p > 0.05, d = 0.40. The independent-sample t-test also showed that the DF benefits under the self-referential condition (M = 0.24, SE = 0.05) were significantly higher than those under the other-referential condition (M = 0.06, SE = 0.06), t (32) = 2.16, p = 0.039, d = 0.74.

**Cued-recall performance:** Cued-recall rates were submitted to a 2 (group: experimental group vs. control group) × 2 (reference: self-reference vs. other-reference) × 2 (item type: TBF/R vs. TBR/R) mixed-factorial ANOVA with a within-subjects factor presentation reference and item type and a between-subjects factor group. The analysis indicated a significant main effect of item type, F (1, 32) = 36.01, p < 0.001, η_p_^2^ = 0.53. The cued-recall rate of TBF/R items (M = 0.29, SE = 0.03) was significantly lower than that of TBR/R items (M = 0.46, SE = 0.03). There was a significant interaction between group and item type, F (1, 32) = 28.70, p < 0.001, η_p_^2^ = 0.47. The simple effect analysis showed that the cued-recall rate of TBF/R items (M = 0.18, SE = 0.04) was significantly lower than that of TBR/R items (M = 0.51, SE = 0.04), p < 0.001, η_p_^2^ = 0.67, in the experimental group, but there was no difference in cued-recall rates between TBF/R items (M = 0.40, SE = 0.04) and TBR/R items (M = 0.42, SE = 0.04) in the control group, p > 0.05. Other main effects and interaction effects were not significant, all Fs < 1. We used pairwise comparisons to compare the costs and benefits of DF under different referential conditions. The result revealed significant DF costs under the self-referential condition [F (1, 32) = 9.14, p < 0.001, η_p_^2^ = 0.22] and the other-referential condition [F (1, 32) = 11.30, p = 0.002, η_p_^2^ = 0.26]. However, DF benefits were not significant under the two referential conditions, ps > 0.05.

To further analyze the data, we conducted an independent-sample t-test with the DF effect under the self-referential condition and the other-referential condition in the experimental group. The result revealed no significant difference between the DF effect under the self-referential condition and the other-referential condition, t (32) = 1.49, p > 0.05, d = 0.51. The independent-sample t-test showed that there was no significant difference between the DF costs under the self-referential condition and the other-referential condition, t (32) = 0.06, p > 0.05, d = 0.02. The independent-sample t-test also revealed no significant difference between the DF benefits under the self-referential condition and the other-referential condition, t (32) = 0.82, p > 0.05, d = 0.28.

**Summary of behavioral analyses:** Behavioral data showed that the instruction to forget induced DF, indicating that our operations were effective. The three-way interaction of free-recall rates showed that there was a DF effect under the self-referential condition in the experimental group. This indicates that self-referential processing cannot prevent the inhibitory effect of the inhibition mechanism on self-referential materials, consistent with some previous studies [8, 9, 14, 15, 17]. However, the three-way interaction also revealed a DF effect under the other-referential condition in the experimental group. Most previous studies have observed DF under a self-reference condition, while the DF effect disappears under an other-reference condition [14, 15, 17]. Some studies have found DF under a self-referential condition and an other-referential condition, but the DF effect under the self-referential condition is weaker [8, 9]. To further explore the differences in DF effects under different referential conditions, we conducted an independent-sample t-test on the behavioral data and found that the DF effect in the self-referential condition was stronger than that in the other-referential condition only in the free-recall test. However, there was no such difference in the cued-recall test. Based on the previously mentioned perspective of inhibition, this result can be interpreted as the self being unique in the level of consciousness of the individual so that self-related information will be encoded in more detail. (The main effect of reference was not insignificant in Experiment 1, and it seems that self-referential processing does not show a memory advantage effect; therefore, we will discuss it at the end of this article.) When receiving DF instructions, participants used some means to separate TBF items and TBR items. Therefore, TBF items and TBR items had higher discrimination due to elaborate encoding, and cognitive resources and rehearsal opportunities were left to TBR items. At the same time, the inhibitory mechanism induced by DF instructions attempted to exclude those TBF items from consciousness, resulting in DF [14]. In the other-referential condition, because the learning materials could not obtain the same unique processing as in the self-referential condition, there was lower discrimination between learning materials [17]. However, we observed the DF effect under the other-referential condition, although this effect was weaker. In addition, due to the presence of cued words in the cued-recall test, the retrieval of target words was facilitated [20], resulting in no difference in the DF effect under the two referential conditions.

We used a method based on the principle of cost-benefit to analyze the data. When learning materials require free recall, the costs and benefits of DF exist in a self-referential condition. We found DF costs only under the other-referential condition, and the DF benefits were significantly higher under the self-referential condition than under the other-referential condition. However, there were only DF costs in the cued-recall test for both the self-referential condition and the other-referential condition, and there was no significant difference in these costs.

The above results indicate that both self-referential processing and other-referential processing can induce DF, while the DF effect under the self-referential condition is stronger, which might be caused by the inhibition mechanism. Moreover, the analysis based on the principle of cost-benefit showed that the costs and benefits of DF in the item method did not exist simultaneously. The DF effect might be the result of multiple mechanisms functioning together. To further examine these assumptions with quantitative analysis, we will use the MPT model to analyze the data.

#### Model data analyses

The goodness of fit of the MPT models is determined by measuring the distance between the model-implied frequencies and the observed category frequencies. PD^λ^ defines an asymptotically χ^2^ distributed family of distance measures, where λ is a family parameter, and the log-likelihood ratio statistic G^2^ is a special case with λ = 0 [35]. If we want to determine whether the model fits the experimental data or whether the values of the same parameter are different under different experimental conditions, we can use log-likelihood ratio statistic G^2^ to test the hypothesis [36] and to determine whether the model fit and the difference between the parameters are statistically significant. In the model test, we obtain the frequencies of the six types of behavior events in different experimental conditions by gathering the experimental data. An MPT model can be built for each experimental condition, and each model contains five parameters (a, r, s, u, and l). There are eight experimental conditions in this study, for a total of 40 parameters. With six observable events per condition, there are (6–1) × 8 = 40 free categories in the data. If the number of free parameters is equal to the number of free categories, the parameters can be estimated, but the model fit cannot be tested [25]. Because the interval between the free-recall test and the cued-recall test is the same and short for each experimental condition, the loss of memory caused by the time delay should be quite low and equal. Thus, we set the l parameter to be equal across all conditions to obtain a testable model with spare degrees of freedom [20]. We also used the software multiTree [35] (Linux, MacOS, and Windows versions of multiTree can be downloaded from http://psycho3.uni-mannheim.de/multitree) to fit this restricted model to the data, which yielded a good fit, G^2^ (7) = 8.15, p = 0.319 (Riefer and Rouder (1992) noted that we can not only set the l parameters between the storage-retrieval MPT models with different conditions to be equal but also set parameter s and parameter u within each model to be equal. This restriction would have resulted in a significantly worse fit, G2 (15) = 29.51, p = 0.013. Therefore, this restriction was not applied here). Next, we will examine the differences between the parameters under different conditions (the frequency of six kinds of behavioral events under each condition is given in Table 1).

**Table 1:** Frequencies of all recall events by Experiments 1.

Condition	E_1_	E_2_	E_3_	E_4_	E_5_	E_6_
**Experimental group** **self-reference**						
TBF/R-items	6	0	0	2	27	169
TBR/R-items	73	5	9	10	28	79
**Experimental group** **other-reference**						
TBF/R-items	12	1	2	6	27	156
TBR/R-items	46	5	11	10	42	90
**Control group** **self-reference**						
TBF/R-items	33	2	9	9	40	90
TBR/R-items	28	3	7	11	49	106
**Control group** **self-reference**						
TBF/R-items	37	0	5	8	41	113
TBR/R-items	38	1	8	11	39	107

E_1_ = both items freely recalled, correct cued recall; E_2_ = both items freely recalled, incorrect cued recall; E_3_ = one item freely recalled, correct cued recall; E_4_ = neither item freely recalled, correct cued recall; E_5_ = one item freely recalled, incorrect cued recall; E_6_ = neither item freely recalled, incorrect cued recall. TBF/R items were items for which participants in the experimental group received “forget” cues, whereas participants in the control group received “remember” cues. For TBR/R items, all participants received “remember” cues.

**Fig 4:**
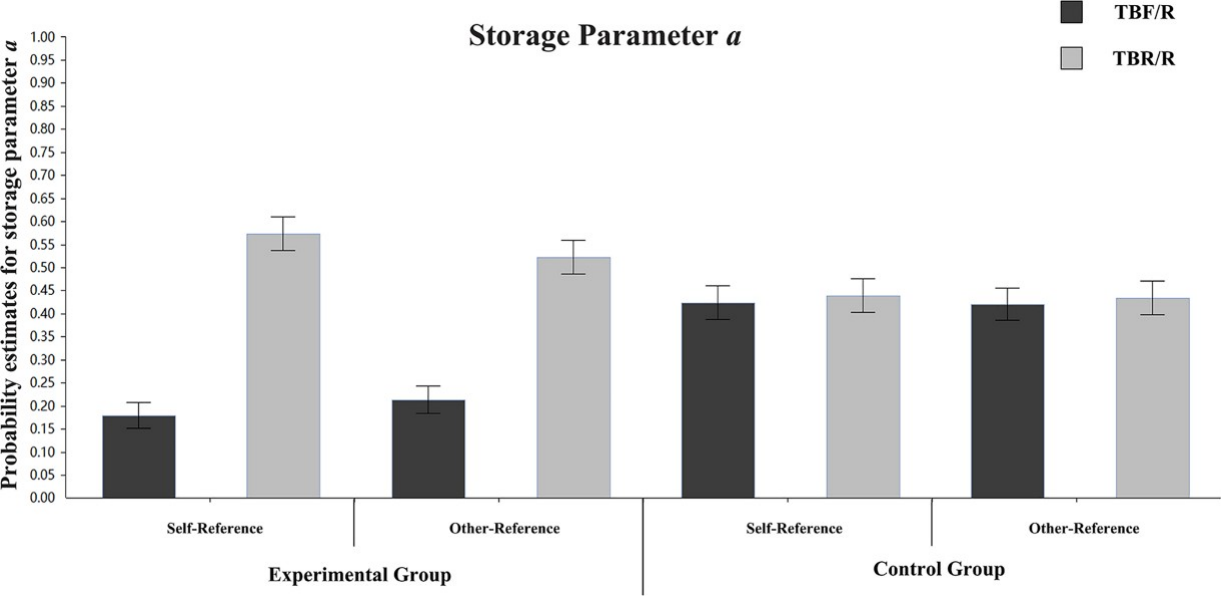
Parameter estimates for the probability of storage parameter a. Error bars depict standard errors.

**Storage parameter a:** To explore the psychological mechanism of DF under different referential conditions, we first compared the differences in storage parameter a between TBF/R items and TBR/R items in different groups and references. The parameter estimation is presented in Fig 4. In the self-referential condition of the experimental group, the differences in storage parameter a between TBF/R items and TBR/R items were significant (a_TBF/R_ = 0.18, a_TBR/R_ = 0.57), ΔG^2^ (1) = 66.12, p < 0.001. In the self-referential condition of the control group, the differences in storage parameter a between TBF/R items and TBR/R items were not significant (a_TBF/R_ = 0.42, a_TBR/R_ = 0.44), ΔG^2^ (1) = 0.09, p > 0.05. In the other-referential condition of the experimental group, the differences in storage parameter a between TBF/R items and TBR/R items were significant (a_TBF/R_ = 0.21, a_TBR/R_ = 0.52), ΔG^2^ (1) = 40.25, p < 0.001. In the other-referential condition of the control group, the differences in storage parameter a between TBF/R items and TBR/R items were not significant (a_TBF/R_ = 0.42, a_TBR/R_ = 0.43), ΔG^2^ (1) = 0.08, p > 0.05. This result indicates that instructions to forget reduce the storage rates of TBF/R items under the self-referential and other-referential conditions.

To illustrate the storage of different types of items under different referential conditions in the experimental group, we compared their storage parameter a. For TBF/R items, there were no significant estimate differences between the self-referential and other-referential conditions (a_self-reference_ = 0.18, a_other-reference_ = 0.21), ΔG^2^ (1) = 0.73, p > 0.05. For TBR/R items, there were no significant estimate differences between the self-referential and other-referential conditions (a_self-reference_ = 0.57, a_other-reference_ = 0.52), ΔG^2^ (1) = 0.98, p > 0.05.

**Fig 5:**
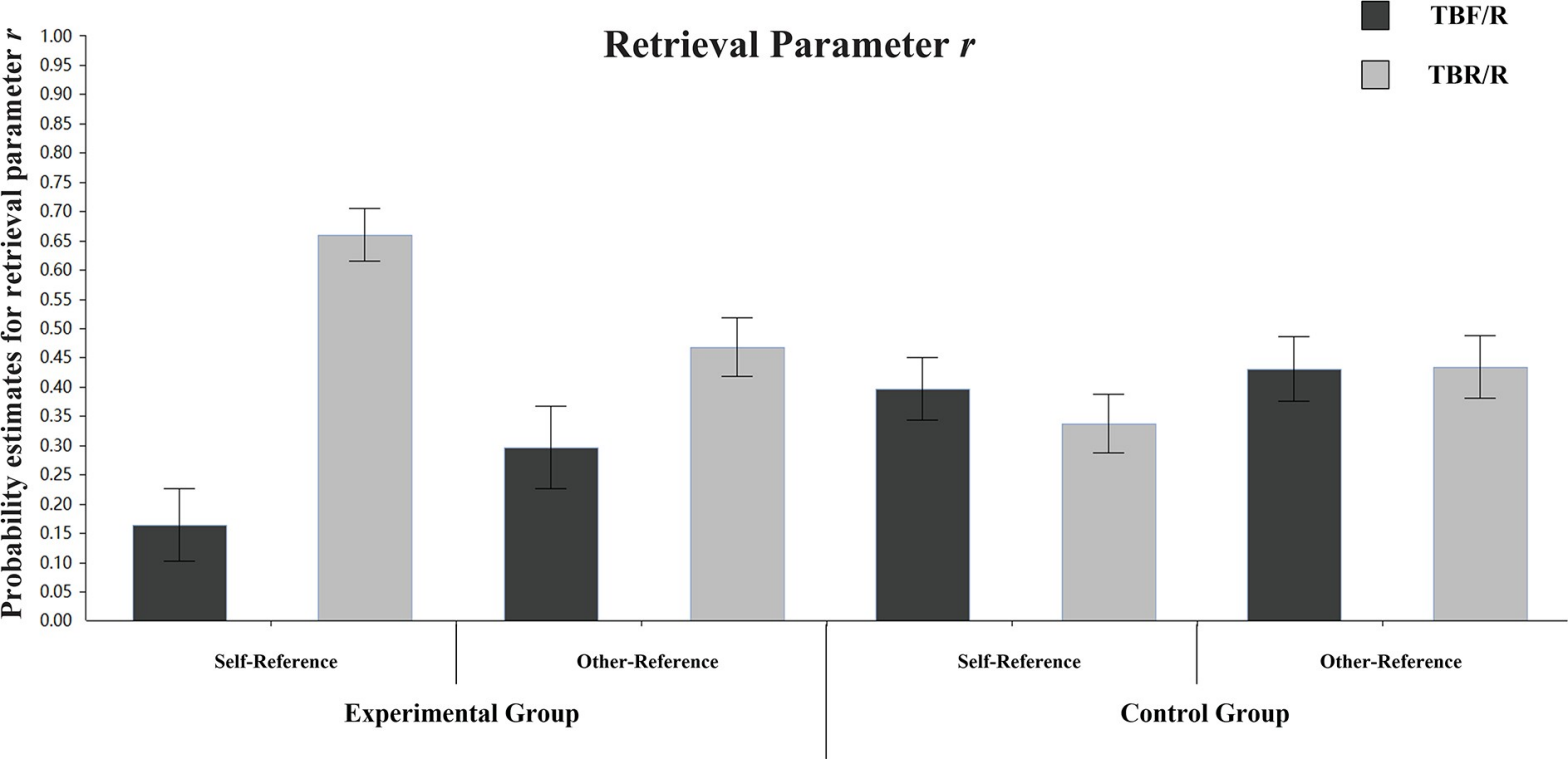
Parameter estimates for the probability of retrieval parameter r. Error bars depict standard errors.

**Retrieval parameter r:** Fig 5 shows the differences in retrieval parameter r between TBF/R items and TBR/R items in different groups and referential conditions. In the self-referential condition of the experimental group, the differences in retrieval parameter r between TBF/R items and TBR/R items were significant (r_TBF/R_ = 0.16, r_TBR/R_ = 0.66), ΔG^2^ (1) = 27.97, p < 0.001. In the self-referential condition of the control group, the differences in retrieval parameter r between TBF/R items and TBR/R items were not significant (r_TBF/R_ = 0.40, r_TBR/R_ = 0.34), ΔG^2^ (1) = 0.62, p > 0.05. In the other-referential condition of the experimental group, the differences in retrieval parameter r between TBF/R items and TBR/R items were marginally significant (r_TBF/R_ = 0.30, r_TBR/R_ = 0.47), ΔG^2^ (1) = 3.69, p = 0.055. In the other-referential condition of the control group, the differences in retrieval parameter r between TBF/R items and TBR/R items were not significant (r_TBF/R_ = 0.43, r_TBR/R_ = 0.43), ΔG^2^ (1) = 0.00, p > 0.05. The result indicates that instructions to forget reduce the retrieval rates of TBF/R items under the self-referential and other-referential conditions.

We then compared retrieval parameter r of different types of items under different referential conditions in the experimental group. For TBF/R items, there were no significant r estimate differences between the self-referential and other-referential conditions (r_self-reference_ = 0.16, r_other-reference_ = 0.30), ΔG^2^ (1) = 1.94, p > 0.05. For TBR/R items, there were significant r estimate differences between the self-referential and other-referential conditions (r_self-reference_ = 0.66, r_other-reference_ = 0.47), ΔG^2^ (1) = 7.77, p = 0.005. These results indicated that the retrieval rate of TBR/R items was higher under the self-referential condition than under the other-referential condition, which was the main reason for the difference in the DF effect.

**Additional parameters s, u, and l:** Parameters s and u measure the singleton recall of a word pair, increasing the model’s validity and eliminating additional interference factors. They indicate that the cognitive states in the standard free-then-cued-recall paradigm occur rather infrequently (see Table 1), so the values are small (i.e., estimates range from 0.00 to 0.14). As expected, the probability of memory loss during the delay between the free and cued recall was very low (l = 0.05, SE = 0.01). Because these parameters were small and not central to our discussion, they can be regarded as “nuisance parameters” [36] without further discussion. For transparency, the probability estimate for all parameters is displayed in S1 Table.

**Summary of model-based analyses:** The model-based results showed that storage parameter a and retrieval parameter r of TBF/R items were significantly lower than those of TBR/R items under both self-referential processing and other-referential processing in the experimental group. In the control group, there were no significant differences between TBF/R and TBR/R items in storage parameters a and retrieval parameter r under self-referential processing or other-referential processing. This finding indicated that TBF and TBR items were psychologically separated during the information encoding phase due to the effect of instructions, and more cognitive resources were distributed to TBR items so that individuals could selectively rehearse and process this part of the material more elaborately [16, 37, 38]. Therefore, TBR items were stored more easily in the memory system. The role of the instruction to forget in the information retrieval phase inhibited previous TBF items; consequently, participants were obstructed or inhibited in retrieving the TBF items, which made TBF item recall performance worse than that of TBR items [39–44]. Therefore, the DF effect under both referential conditions was the result of both selective rehearsal and retrieval inhibition working together, supporting H3. The test of the differences between the TBF/R items and TBR/R items in parameter a and parameter r under different referential conditions in the experimental group showed that parameter a of the two types of items did not differ significantly between referential conditions. However, under the self-referential condition, parameter r of TBR/R items was significantly higher than that of TBR/R items under the other-referential condition. The results showed that the encoding and storage of the learning materials under different referential conditions did not cause differences in the DF effect. The main reason for this difference was that the retrieval rate of TBR/R items under the self-referential condition was higher than that of TBR/R items under the other-referential condition. This finding is not entirely consistent with the focus of Li et al. (2005) on the use of the inhibition mechanism to explain DF under the self-referential condition or our H2.

However, based on existing studies, we find that there are three main ways to induce self- or other-reference: (1) Participants are asked to determine the degree of conformity of trait adjectives and emotional adjectives with themselves or others [9, 14, 16, 17]. (2) Participants are asked to imagine themselves or others in a certain state [15]. (3) Participants are asked to imagine that something is their own or someone else’s [8]. These referential methods directly indicate what type of state participants or other people are in and distinguish themselves clearly from others, thus reflecting an explicit reference effect. Explicit and intentional self-referential processing is relatively unusual in daily life. In most social contexts, self-referential processing usually involves relatively automatic associations between the self and external stimuli [45–47]. For example, when a courageous person sees someone fall into a river, he or she does not think, “I should save the drowning person because I am a warmhearted person” or “I should save the drowning person because I can swim.” Instead, the behavior is guided by an incidental unconscious self-referential processing approach [48]. Likewise, incidental other-referential processing often occurs automatically. For example, an individual may unintentionally see a colleague putting a key into a drawer. Later, the colleague may forget where he put the key. When asked, the individual may remember that the key is in the drawer. However, at the initial time, the individual did not care and did not think about it deliberately (i.e., he/she did not think that the colleague put the key into the drawer and may forget it, so he/she should help the colleague remember it). We regard these types of self- or other-referential processing as implicit referential processing. Can such implicit referential processing cause DF? If so, is there a difference in the DF effects between different referential conditions, and are the results consistent with explicit referential processing results? We will discuss these questions in Experiment 2.

## Experiment 2

The purpose of Experiment 2 is to explore whether implicit self-reference has an impact on DF, whether the influence of implicit self-reference on DF differs from the influence of implicit other-reference, and what the psychological mechanism is that causes this impact. Incidental self-processing is an implicit and unconscious self-referential processing propensity. Because the connection between the self and external stimuli often occurs automatically and incidental self-processing is an important manifestation of this automatic connection, we use tasks that can induce incidental self-processing to improve ecological validity [48]. Zhu et al. (2013) proposed that participants reacted more quickly to their own handwriting than to other people’s handwriting, demonstrating that handwriting can induce incidental self-processing [49]. Turk et al. (2008) used names and faces to examine the effect of incidental self-processing on memory and found that memory of self-related materials is better than memory of other-related materials. Therefore, Experiment 2 used handwriting to induce incidental self-referential processing and other-referential processing to investigate the DF effect under implicit referential conditions. We propose two hypotheses:

H4. Implicit self-referential information impairs the DF effect, supporting the perspective of encoding.

H5. Implicit self-referential information enhances the DF effect, supporting the perspective of inhibition.

### Method

#### Ethics statement

The study was approved by the local ethics committee of Hebei University in China. All participants provided verbal informed consent prior to inclusion in the study.

#### Participants

Thirty students from a university in Hebei province (24 women; M_age_ = 23.83 years, SD_age_ = 1.56, range: 20–27 years) participated in this experiment. All participants had normal intelligence and normal or corrected-to-normal vision and reported no current or past neurological or psychiatric disease. Each participant received monetary compensation and small gifts after the experiment.

#### Materials

Experiment 2 used the same materials as Experiment 1. To induce incidental self-processing and other-processing, we randomly divided the learning materials according to the group, the matching of cue words and target words, the reference of word pairs (i.e., self or other), and the instructions followed by word pairs. Then, the self-referential items to be learned by each participant were divided according to a single Chinese character, and the order was disrupted (after the split, the meaning of the single Chinese character was not related to the meaning of the original word). The participants used a 0.5 mm black gel pen for transcription. A psychology graduate student transcribed all 152 two-character Chinese nouns that were chosen as referential materials for each participant according to the different conditions. To prevent the participants from guessing the aim of the experiment, we distributed the materials to participants through a middleman and asked them to complete the Chinese version of the Transgression-Related Interpersonal Motivations Scale-12-Item Form (TRIM-12) [50] before transcribing the materials. The experimental materials were produced by first collecting all the materials that participants had transcribed and scanning them together with other-referential materials into a PDF format. Foxit Reader software was used to open the PDF files and to intercept a single Chinese character using the screenshot function. We then used Visio 2010 software to splice single Chinese characters into the previously selected two-character Chinese nouns (at a uniform size of 1 inch wide × 0.6 inches tall). Finally, the two-character Chinese nouns were matched according to cue-target word pairs and placed on a black background in white font at a resolution of 1920 × 1080 as JPG format images.

#### Design

The design was a 2 (group: experimental group vs. control group) × 2 (reference: self-reference vs. other-reference) × 2 (item type: TBF/R vs. TBR/R) mixed design with a within-subjects factor presentation reference and item type and a between-subjects factor group. Experiment 2 used the same paradigm, grouping mode, and item type as Experiment 1.

#### Procedure

**Fig 6:**
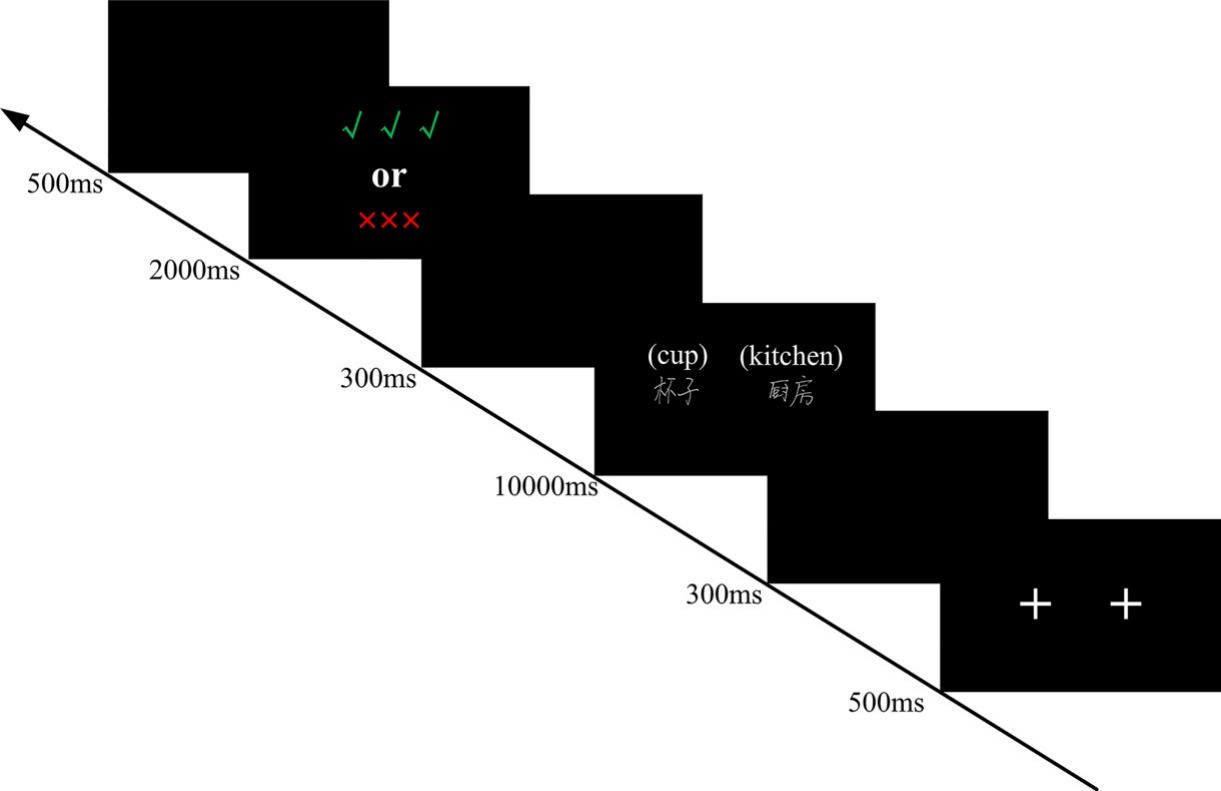
The Procedure of Experiment 2.

The experimental procedure for each participant was written and run separately using E-prime 2.0 software based on the previously identified groupings and their respective experimental materials. The experimental materials were presented on a Dell 23.8-inch LED monitor with a screen resolution of 1920 × 1080 with a black background. Experiment 2 was divided into four phases. These differed from Experiment 1 in that it was not necessary to use a sentence to induce self-reference or other-reference; it was only necessary to remember the word pairs. Participants were able to experiment until one month after they had transcribed the experimental materials. To make the learning time of the material consistent with that of Experiment 1, we set the presentation time of the word pairs to 10000 ms. The other processes were consistent with those of Experiment 1 (see Fig 6).

### Results and discussion

#### Behavioral data analyses

**Fig 7:**
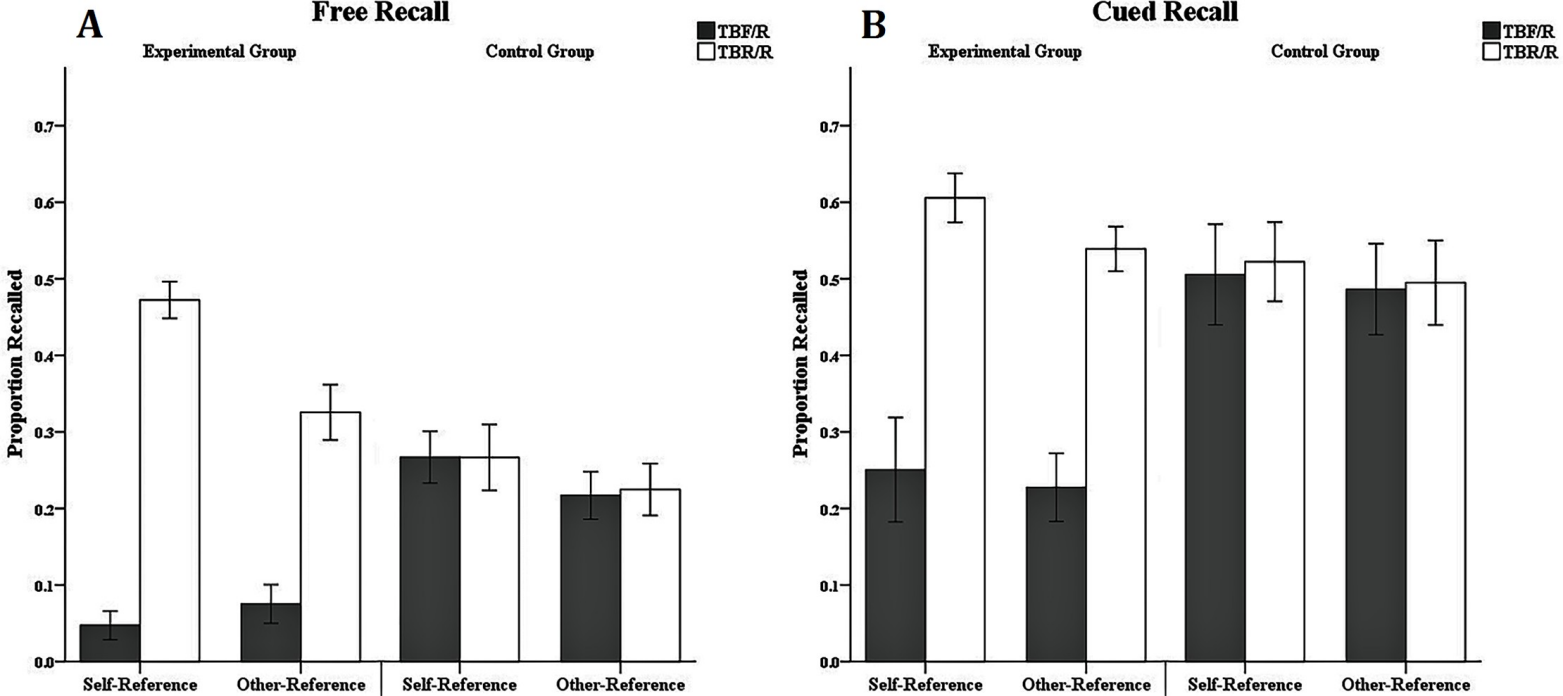
Mean proportion of TBF/R-item and TBR/R-item recall in Experiment 2. Mean proportion of TBF/R-item and TBR/R-item recall as a function of referential categories (self-reference, other-reference), group categories (experimental group, control group) and memory instruction (forget, remember) for free recall (Fig 7A) and cued recall (Fig 7B) in Experiment 2. Error bars depict standard errors.

Fig 7 shows the results of the ANOVA with the dependent variables of the free-recall rates of the word pairs and the cue recall rates of target words under different referential conditions.

**Free-recall performance:** Free-recall rates were submitted to a 2 (group: experimental group vs. control group) × 2 (reference: self-reference vs. other-reference) × 2 (item type: TBF/R vs. TBR/R) mixed-factorial ANOVA with a within-subjects factor presentation reference and item type and a between-subjects factor item type. The analysis indicated no main effect of group, F (1, 28) = 0.29, p > 0.05. There was no difference in free-recall rates between the experimental group (M = 0.23, SE = 0.02) and the control group (M = 0.24, SE = 0.02). The analysis indicated a significant main effect of reference, F (1, 28) = 6.07, p = 0.020, η_p_^2^ = 0.18. The free-recall rate of the self-referential materials (M = 0.26, SE = 0.02) was significantly higher than that of the other-referential materials (M = 0.21, SE = 0.02). There was a significant main effect of item type, F (1, 28) = 50.60, p < 0.001, η_p_^2^ = 0.64. The free-recall rate of TBF/R items (M = 0.15, SE = 0.02) was significantly lower than that of TBR/R items (M = 0.32, SE = 0.02). The interaction between group and reference was not significant, F (1, 28) = 0.10, p > 0.05. There was a significant interaction between group and item type, F (1, 28) = 48.32, p < 0.001, η_p_^2^ = 0.63. The simple effect analysis showed that the free-recall rate of TBF/R items (M = 0.06, SE = 0.02) was significantly lower than that of the TBR/R items (M = 0.40, SE = 0.03), p < 0.001, η_p_^2^ = 0.78, in the experimental group, but there was no difference in free-recall rates between TBF/R items (M = 0.24, SE = 0.02) and TBR/R items (M = 0.25, SE = 0.03) in the control group, p > 0.05. There was a significant interaction between reference and item type, F (1, 28) = 5.51, p = 0.026, η_p_^2^ = 0.16. The simple effect analysis showed that the free-recall rate of TBF/R items (M = 0.16, SE = 0.02) in the self-referential conditions was significantly lower than that of the TBR/R items (M = 0.37, SE = 0.03), p < 0.001, η_p_^2^ = 0.62, and the free-recall rate of TBF/R items (M = 0.15, SE = 0.02) in the other-referential conditions was also significantly lower than that of the TBR/R items (M = 0.28, SE = 0.03), p < 0.001, η_p_^2^ = 0.43. The three-way interaction effect between group, reference, and item type was significant, F (1, 28) = 6.65, p = 0.015, η_p_^2^ = 0.19. We conducted pairwise comparisons for the analysis of the free-recall rates and found that the free-recall rate of TBF/R items (M = 0.05, SE = 0.03) in the experimental group under the self-referential condition was significantly lower than that of the TBR/R items (M = 0.47, SE = 0.04), p < 0.001, η_p_^2^ = 0.76. Additionally, the free-recall rate of TBF/R items (M = 0.08, SE = 0.03) was significantly lower than that of TBR/R items under the other-referential condition (M = 0.33, SE = 0.04), p < 0.001, η_p_^2^ = 0.59. There was no significant difference between the free-recall rates of TBF/R items and TBR/R items in the control group in either the self-referential or the other-referential condition, Fs < 1. We used pairwise comparisons to compare the costs and benefits of DF under different referential conditions. The result indicated significant DF costs [F (1, 28) = 17.40, p < 0.001, η_p_^2^ = 0.38] and DF benefits [F (1, 28) = 17.40, p < 0.001, η_p_^2^ = 0.38] under the self-referential condition. Under the other-reference condition, DF costs were significant [F (1, 28) = 12.56, p = 0.001, η_p_^2^ = 0.31], and DF benefits were marginally significant [F (1, 28) = 4.11, p = 0.052, η_p_^2^ = 0.13].

To further analyze the data, we conducted an independent-sample t-test with the DF effect under the self-referential condition and the other-referential condition in the experimental group. The result indicated that the DF effect (M = 0.42, SE = 0.03) under the self-referential condition was stronger than the DF effect (M = 0.25, SE = 0.04) under the other-referential condition, t (28) = 3.30, p = 0.003, d = 1.20. The independent-sample t-test revealed no significant difference in DF costs between the self-referential condition and the other-referential condition, t (28) = 1.34, p > 0.05, d = 0.49. The independent-sample t-test revealed no significant difference in DF benefits between the self-referential condition and the other-referential condition, t (28) = 1.57, p > 0.05, d = 0.57.

**Cued-recall performance:** Cued-recall rates were submitted to a 2 (group: experimental group vs. control group) × 2 (reference: self-reference vs. other-reference) × 2 (item type: TBF/R vs. TBR/R) mixed-factorial ANOVA with a within-subjects factor presentation reference and item type and a between-subjects factor item type. The analysis indicated a significant main effect of item type, F (1, 28) = 36.69, p < 0.001, η_p_^2^ = 0.57. The cued-recall rate of TBF/R items (M = 0.37, SE = 0.04) was significantly lower than that of TBR/R items (M = 0.54, SE = 0.03). There was a significant interaction between group and item type, F (1, 28) = 31.51, p < 0.001, η_p_^2^ = 0.53. The simple effect analysis showed that the cued-recall rate of TBF/R items (M = 0.24, SE = 0.06) was significantly lower than that of the TBR/R items (M = 0.57, SE = 0.04), p < 0.001, η_p_^2^ = 0.71, in the experimental group, but there was no difference in cued-recall rates between TBF/R items (M = 0.50, SE = 0.06) and TBR/R items (M = 0.51, SE = 0.04) in the control group, p > 0.05. Other main effects and interaction effects were not significant, all Fs < 1. We used pairwise comparisons to compare the costs and benefits of DF under different referential conditions. The result revealed significant DF costs under the self-referential condition [F (1, 28) = 7.24, p = 0.012, η_p_^2^ = 0.21] and the other-referential condition [F (1, 28) = 12.16, p = 0.002, η_p_^2^ = 0.30]. However, DF benefits were not significant under the two referential conditions, ps > 0.05.

To further analyze the data, we conducted an independent-sample t-test with the DF effect under the self-referential condition and the other-referential condition in the experimental group. The result indicated no significant difference in the DF effect between the self-referential condition and the other-referential condition, t (28) = 0.61, p > 0.05, d = 0.10. The independent-sample t-test showed that there was no significant difference in DF costs between the self-referential condition and the other-referential condition, t (28) = 0.03, p > 0.05, d = 0.01. The independent-sample t-test also showed no significant difference in the DF benefits between the self-referential condition and the other-referential condition, t (28) = 0.43, p > 0.05, d = 0.16.

**Summary of behavioral analyses:** The behavioral data showed that the instruction to forget induced DF, indicating that our operations were effective. The experimental group showed a DF effect under the self-referential condition, and the recall rates of the self-referential materials were significantly higher than those of the other-referential materials. This result indicated that handwriting-induced incidental self-processing could also cause DF, although this implicit self-referential processing has a memory advantage effect. Moreover, the experimental group showed a DF effect under the other-referential condition. We conducted an independent-sample t-test and found that the DF effect in the self-referential condition was stronger than that in the other-referential condition only in the free-recall test. There was no such difference in the cued-recall test.

We used a method based on the principle of cost-benefit to analyze the data. When learning materials required free recall, the costs and benefits of DF existed in the self-referential condition, and there were DF costs and marginally significant DF benefits under the other-referential condition. Furthermore, the costs and benefits of DF did not differ between the two referential conditions. There were DF costs only in the cued-recall test for both the self-referential condition and the other-referential condition, and there was no significant difference in these costs.

The above results are almost consistent with those of Experiment 1. It can be confirmed that both incidental self-referential processing and incidental other-referential processing induce DF, and the DF effect under the incidental self-referential condition is stronger. The differences from Experiment 1 were that implicit self-referential processing showed a memory advantage effect, and the experimental group in Experiment 2 showed marginally significant DF benefits under the other-reference condition. Is the DF effect under the condition of implicit self-reference or other-reference the result of multiple mechanisms functioning together? Are the reasons for the difference in the DF effect under different referential conditions the same as in Experiment 1 or the same as in previous studies? We will use the MPT model to further analyze the data.

#### Model data analyses

We set the l parameter to be equal across all conditions and used the software multiTree to fit this restricted model to the data, which yielded a good fit, G^2^ (7) = 7.40, p > 0.05. According to the method proposed by Riefer and Rouder (1992), we further set the s parameter and u parameter within each model to be equal. In this condition, the restricted model also yielded a good fit, G^2^ (15) = 17.77, p > 0.05, and there was no difference in the degree of fit of the former restricted model, ΔG^2^ (8) = 10.37, p > 0.05. Therefore, we used the simplest model for the subsequent data analyses (the frequency of six types of behavioral events under each condition is given in Table 2).

**Table 2:** Frequencies of all recall events by Experiments 2.

Condition	E_1_	E_2_	E_3_	E_4_	E_5_	E_6_
**Experimental group** **self-reference**						
TBF/R-items	9	0	0	1	38	132
TBR/R-items	74	3	4	12	31	56
**Experimental group** **other-reference**						
TBF/R-items	9	0	3	4	29	135
TBR/R-items	47	4	6	9	44	70
**Control group** **self-reference**						
TBF/R-items	42	0	5	7	44	82
TBR/R-items	41	2	3	6	51	77
**Control group** **self-reference**						
TBF/R-items	34	1	5	3	49	88
TBR/R-items	30	3	4	11	56	76

E_1_ = both items freely recalled, correct cued recall; E_2_ = both items freely recalled, incorrect cued recall; E_3_ = one item freely recalled, correct cued recall; E_4_ = neither item freely recalled, correct cued recall; E_5_ = one item freely recalled, incorrect cued recall; E_6_ = neither item freely recalled, incorrect cued recall. TBF/R items were items for which participants in the experimental group received “forget” cues, whereas participants in the control group received “remember” cues. For TBR/R items, all participants received “remember” cues.

**Fig 8:**
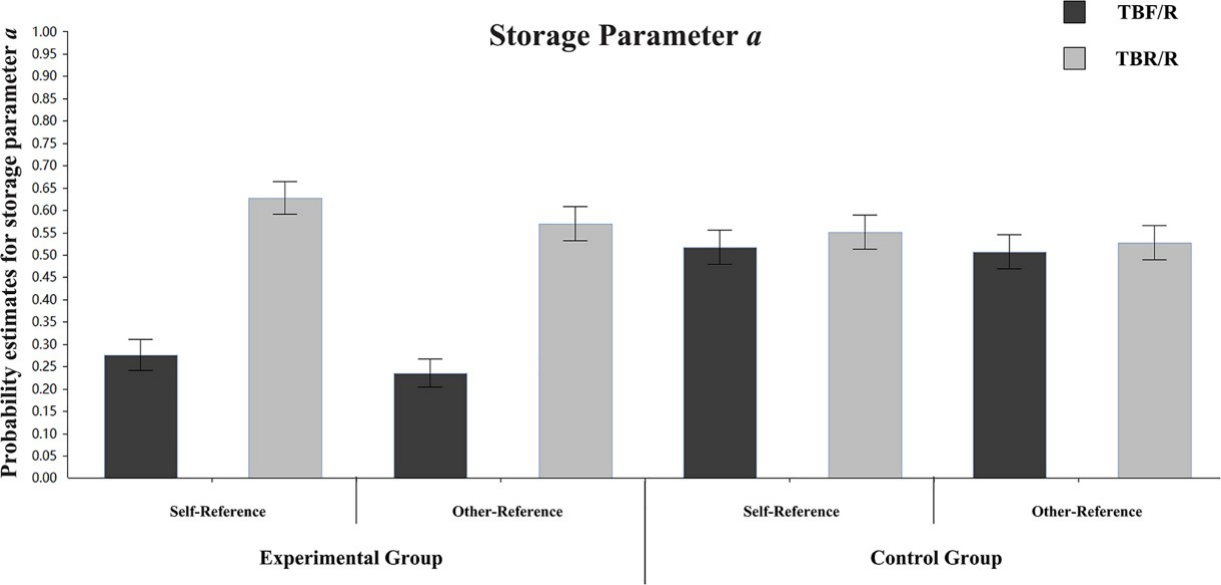
Parameter estimates for the probability of storage parameter a. Error bars depict standard errors.

**Storage parameter a:** To explore the psychological mechanism of DF under different referential conditions, we first compared the differences in storage parameter a between TBF/R items and TBR/R items in different groups and references. The parameter estimation is presented in Fig 8. In the self-referential condition of the experimental group, the differences in storage parameter a between TBF/R items and TBR/R items were significant (a_TBF/R_ = 0.28, a_TBR/R_ = 0.63), ΔG^2^ (1) = 44.08, p < 0.001. In the self-referential condition of the control group, the differences in storage parameter a between TBF/R items and TBR/R items were not significant (a_TBF/R_ = 0.52, a_TBR/R_ = 0.55), ΔG^2^ (1) = 0.39, p > 0.05. In the other-referential condition of the experimental group, the differences in storage parameter a between TBF/R items and TBR/R items were significant (a_TBF/R_ = 0.23, a_TBR/R_ = 0.57), ΔG^2^ (1) = 40.75, p < 0.001. In the other-referential condition of the control group, the differences in storage parameter a between TBF/R items and TBR/R items were not significant (a_TBF/R_ = 0.51, a_TBR/R_ = 0.53), ΔG^2^ (1) = 0.15, p > 0.05. These results indicate that the instruction to forget reduces the storage rates of TBF/R items under incidental self-referential and incidental other-referential conditions.

To illustrate the storage of different types of items under different referential conditions in the experimental group, we compared their storage parameter a. For TBF/R items, there were no significant estimate differences between the self-referential and other-referential conditions (a_self-reference_ = 0.27, a_other-reference_ = 0.23), ΔG^2^ (1) = 0.74, p > 0.05. For TBR/R items, there were no significant estimate differences between the self-referential and other-referential conditions (a_self-reference_ = 0.63, a_other-reference_ = 0.57), ΔG^2^ (1) = 1.23, p > 0.05.

**Fig 9:**
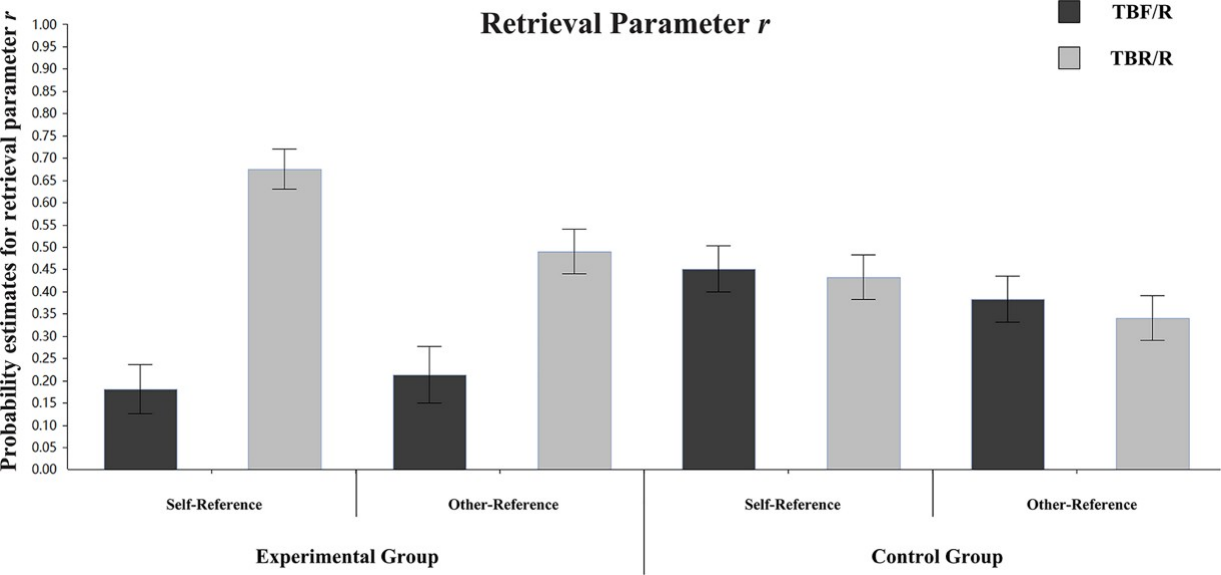
Parameter estimates for the probability of retrieval parameter r. Error bars depict standard errors.

**Retrieval parameter r:** Fig 9 shows the differences in retrieval parameter r between TBF/R items and TBR/R items in different groups and referential conditions. In the self-referential condition of the experimental group, the differences in retrieval parameter r between TBF/R items and TBR/R items were significant (r_TBF/R_ = 0.18, r_TBR/R_ = 0.68), ΔG^2^ (1) = 34.75, p < 0.001. In the self-referential condition of the control group, the differences in retrieval parameter r between TBF/R items and TBR/R items were not significant (r_TBF/R_ = 0.45, r_TBR/R_ = 0.43), ΔG^2^ (1) = 0.06, p > 0.05. In the other-referential condition of the experimental group, the differences in retrieval parameter r between TBF/R items and TBR/R items were marginally significant (r_TBF/R_ = 0.21, r_TBR/R_ = 0.49), ΔG^2^ (1) = 9.90, p = 0.002. In the other-referential condition of the control group, the differences in retrieval parameter r between TBF/R items and TBR/R items were not significant (r_TBF/R_ = 0.38, r_TBR/R_ = 0.34), ΔG^2^ (1) = 0.36, p > 0.05. The results indicate that the instruction to forget reduced the retrieval rates of TBF/R items under the incidental self-referential and incidental other-referential conditions.

We then compared the retrieval parameter r of different types of items under different referential conditions in the experimental group. For TBF/R items, there were no significant r estimate differences between the self-referential and other-referential conditions (r_self-reference_ = 0.18, r_other-reference_ = 0.21), ΔG^2^ (1) = 0.14, p > 0.05. For TBR/R items, there were significant r estimate differences between the self-referential and other-referential conditions (r_self-reference_ = 0.68, r_other-reference_ = 0.49), ΔG^2^ (1) = 7.36, p = 0.001. These results indicated that the retrieval rate of TBR/R items under the self-reference condition was higher than that of TBR/R items under the other-reference condition, which was the main reason for the difference in the DF effect.

**Additional parameters s, u, and l:** Consistent with Experiment 1 and our expectations, the values of parameters s and u were small (i.e., estimates ranged from 0.00 to 0.10), and the probability of memory loss during the delay between the free and cued recall was very low (l = 0.04, SE = 0.01). Therefore, we do not discuss these three parameters. The probability estimate for all parameters is displayed in S1 Table.

**Summary of model-based analyses:** The model-based results were identical to those of Experiment 1 and showed that storage parameter a and retrieval parameter r of TBF/R items were significantly lower than those of TBR/R items under both self-referential processing and other-referential processing in the experimental group. In the control group, there were no significant differences between TBF/R and TBR/R items in storage parameters a and retrieval parameter r under self-referential processing or other-referential processing. This result indicated that TBF and TBR items were psychologically separated during the information encoding phase due to the effect of instructions and that cognitive resources were distributed more to TBR items so that individuals could selectively rehearse and process this part of the material more elaborately. Therefore, TBR items were stored more easily in the memory system. The role of the instruction to forget in the information retrieval phase inhibited previous TBF items so that participants were obstructed or inhibited from retrieving the TBF items, which made their recall performance of TBF items worse than that of TBR items. Therefore, the DF effect under both implicit referential conditions was the result of selective rehearsal and retrieval inhibition working together. The test of the differences between the TBF/R items and TBR/R items in parameter a and parameter r under different referential conditions in the experimental group showed that parameter a of the two types of items did not differ significantly between the referential conditions. However, parameter r of TBR/R items was significantly higher under the self-referential condition than under the other-referential condition. The results showed that the encoding and storage of learning materials under different implicit referential conditions did not cause differences in the DF effect. The main reason for this difference was that the retrieval rate of TBR/R items under the self-referential condition was higher than that of TBR/R items under the other-referential condition. This finding is not entirely consistent with the findings of Li et al. (2005), which focused on using the inhibition mechanism to explain DF under the self-referential condition, or with our H5.

## General discussion

In this study, the item-method DF paradigm and the storage-retrieval MPT model were combined to examine whether DF occurs under a self-reference condition, what the internal psychological mechanisms of this DF are, whether the DF effect differs between self-referential and other-referential conditions, and what causes this difference. Experiment 1 used an explicit referential processing method in which participants performed self- or other-referential processing through silent reading and imagining. Experiment 2 used an ecological validity method of incidental self-processing to induce implicit self- or other-referential processing through handwriting.

### Internal mental mechanisms of item-method directed forgetting

To exclude the interference of situational factors and to make the experiment more closely aligned to daily life when studying DF encoding and retrieval processes, we used related two-character Chinese nouns as the experimental materials and the item-method DF paradigm as the experimental paradigm. The behavioral data results of Experiments 1 and 2 all indicated that participants had DF under different referential conditions, both in explicit referential processing and in implicit referential processing. In previous studies, researchers used one mechanism of selective rehearsal and retrieval inhibition to explain the internal mental processes of DF. Woodward and Bjork (1971) found that a delay in the presentation of instructions did not affect the DF effects. It was found that participants only conducted maintenance rehearsal on learning materials without deep encoding before instructions were presented and TBR items were selectively rehearsed after instructions were presented, causing the DF effect. Zack et al. (1996) found that the inhibitory control abilities of the elderly deteriorated, and inhibiting the retrieval of TBF items was difficult. Thus, the DF effects were weaker than the DF effects among young people, which supports the retrieval inhibition account. We used a method based on the principle of cost-benefit to analyze the data and found that there were DF costs but no DF benefits in some conditions. This result was inconsistent with the description of a one-mechanism account [51, 52]. Therefore, we suggest that item-method DF might be caused by these two types of mechanisms working together.

Further parameter analyses showed that storage parameter a or retrieval parameter r of TBF/R items in the experimental group, regardless of self-referential processing or other-referential processing, was significantly lower than those of TBR/R items in the experimental group in both Experiment 1 and Experiment 2. In the control group, regardless of whether self-referential processing or other-referential processing was used, storage parameter a and retrieval parameter r were not significantly different between TBR/R items and TBF/R items. This finding indicated that TBF and TBR items were psychologically separated during the information encoding phase due to the effect of instructions and that cognitive resources were distributed more to TBR items so that individuals could selectively rehearse and process this part of the material more elaborately [16, 37, 38]. Therefore, these items were stored more easily in the memory system. In the information retrieval phase, the instruction to forget inhibited previous TBF items; consequently, participants were obstructed or inhibited from retrieving the TBF items, which made their recall performance of TBF items worse than that of TBR items [39–44]. Therefore, the item-method DF effect is the result of selective rehearsal and retrieval inhibition working together. This conclusion has also been supported by previous studies [20, 53–55].

### Differences in the directed forgetting effect under different referential conditions

The results of Experiments 1 and 2 both indicated that participants in the experimental group appeared to have a DF effect under the self-referential condition, which demonstrated that self-referential processing cannot prevent the inhibitory effect of the inhibition mechanism on self-referential materials and causes DF. This result is consistent with those of some previous studies [8, 9, 14, 15, 17]. Similarly, participants in the experimental group demonstrated DF under the other-referential condition. The results of the independent-sample t-test showed that the DF effect under the self-referential condition was stronger than the effect under the other-referential condition in the free-recall test.

However, most previous studies found that DF occurred under the self-referential condition, but this DF effect disappeared under the other-referential condition [14, 15, 17]. These studies noted that the enhancement of the DF effects under the self-referential condition can be interpreted as follows: individuals more elaborately encode information that is associated with the self at the level of consciousness. In the course of the experiment, the participants used some means to distinguish between TBF items and TBR items. Self-related TBF items and TBR items had higher discrimination due to the elaborate encoding. Therefore, TBR items used more cognitive resources and rehearsal opportunities, while TBF items were forgotten by the instruction-induced inhibition mechanism and were excluded from the memory system, causing DF. However, under the other-reference condition, learning materials could not be uniquely processed as in the self-reference condition. Therefore, discrimination between learning materials was low, which led to a non-significant effect of DF. While some studies have found that DF occurs under both self-referential and other-referential conditions, the DF effect under the self-referential condition is smaller [8, 9]. These studies suggested that the diminished DF effect in the self-referential condition could be explained by participants’ greater activity in encoding self-related information, including the use of elaborate and organized memory strategies and improved subsequent retrieval of memory content [7, 13]. This may also enhance the encoding of TBF items [8], resulting in a significant increase in the recall of TBF items under the self-referential condition and impairing the DF effect [9].

To determine the reason for this difference, we tested the differences in the parameters and found that in both Experiments 1 and 2, whether under self-referential or other-referential processing in the experimental group, the encoding and storage of learning materials does not cause differences in the DF effect. The main reason for this difference was that the retrieval rate of TBR/R items was higher under the self-referential condition than under the other-referential condition. This result is not entirely consistent with the view that focuses on using the inhibition mechanism to explain DF under the self-referential condition. Li et al. (2005) proposed that under a self-referential condition, TBF items and TBR items have higher discrimination due to the elaborate encoding and that TBR items obtain more cognitive resources and rehearsal opportunities. TBF items are forgotten by the instruction-induced inhibition mechanism and are excluded from the memory system, causing DF. Under the other-reference condition, learning materials cannot be uniquely processed as in the self-reference condition. Therefore, discrimination between learning materials is low, which leads to a non-significant effect of DF [17]. From this perspective, we should not observe the DF phenomenon under the other-referential condition in the experimental group, and storage parameter a of TBF/R items under the self-referential condition should be significantly lower than that of TBF/R items under the other-referential condition. However, our results showed that the DF effect under the other-referential condition in the experimental group still existed, but it was weaker than the DF effect under the self-referential condition. The reason for this difference was that retrieval parameter r of the TBR/R items was significantly higher under the self-referential condition than under the other-referential condition. This finding indicates that there was no difference in the storage rates of TBF/R items between the self-referential condition and the other-referential condition after the instruction to forget, suggesting that TBF/R items have the same degree of encoding. Similarly, there was no difference in the storage rates of TBR/R items between the self-referential condition and the other-referential condition, and TBF/R items had the same degree of encoding. During the retrieval phase, there was no difference in the retrieval rates of TBF/R items between the self-referential condition and the other-referential condition, but the retrieval rate of TBR/R items under the self-referential condition was significantly higher than that of TBR/R items under the other-referential condition. In other words, although both the inhibition mechanism and the encoding mechanism led to DF, the inhibition effect under the two referential conditions was the same and did not cause different DF effects. This difference is due to the participants retrieving more self-referential TBR/R items.

These results challenge the assumption that inhibition mechanisms can be used to interpret the enhancement of DF effects under a self-referential condition. We present a more reasonable explanation for these results by adjusting and supplementing previous views that good memory is selective. Participants need to distinguish between information related to the self and others and to remember the information they need to remember. At the same time, they must ignore and inhibit information that needs to be forgotten. When the instruction to forget appears, participants use some means to distinguish between TBF items and TBR items. TBF items and TBR items have higher discrimination due to memory selectivity, and TBR items obtain more cognitive resources and rehearsal opportunities. When items are retrieved, TBF items are indistinctively excluded from consciousness by the retrieval mechanism as much as possible. However, participants improve the retrieval of TBR items due to the discrimination and cognitive advantage of self-reference effects [7, 13]. Therefore, the DF effect is stronger under the self-referential condition than under the other-reference condition. In the control group, participants were required to remember a total of 96 two-character Chinese nouns throughout the experiment. This caused memory overload (48 two-character Chinese nouns in the experimental group) and resulted in decreased cognitive control ability [56]. Therefore, participants could not distinguish between self-referential items and other-referential items well and did not show the advantage of retrieval.

### Explicit self-reference processing and implicit self-reference processing

Our research showed that the DF of explicit self-referential processing and implicit self-referential processing had the same internal psychological mechanisms and that the internal psychological mechanisms that caused DF effects differed between self-referential processing and other-referential processing. Rameson et al. (2010) used the fMRI technique and found that explicit self-referential processing and implicit self-referential processing evoked many of the same brain areas; this finding shows that they had the same cognitive processes and supports our experimental results. In addition, we found that explicit self-referential processing did not exhibit a memory advantage effect, but implicit self-referential processing did. Some researchers have noted that when people are thinking about their own experiences, they can “take a step back” and evaluate their own experiences as an “observer” [57]. Kross and Ayduk (2011) found that people can process self-related information from two perspectives. One is a self-immersed perspective, and the other is a self-distanced perspective [58]. When processing information with a self-immersed perspective, individuals process self-related information from the subject using a first-person approach, which may lead to more self-related experience and promote the emergence of a self-referential memory advantage effect. When processing information with a self-distanced perspective, individuals evaluate self-related information as an “observer” [58–60]; they pay less attention to detailed information and construct information by increasing insight and integration with information [60]. From this self-distanced perspective, the self-referential memory advantage effect is reduced [57]. In Experiment 1, we induced explicit self-reference by having participants silently read a sentence and imagine the scene depicted in that sentence. This process of imagining one’s own experiences might be based on a self-distanced perspective that involves treating oneself as an “observer” to evaluate one’s own experiences, so the self-referential memory advantage effect is reduced. Because the learning materials we used were nouns, the self-referential effect was greatly impaired when emotional adjectives or trait adjectives were replaced by non-evaluative nouns [13]. Hence, explicit self-referential processing did not exhibit a memory advantage effect. Implicit self-referential processing had a memory advantage effect, which demonstrated that the incidental self-processing we induced with handwriting was effective. Under the implicit reference condition, participants did not use silent reading or imagination, and the processing of self-related information (e.g., handwriting) was completely in the first-person self-immersed perspective. Therefore, the self-related experience was stronger. Therefore, implicit self-referential processing exhibits a memory advantage effect.

These results indicate that self-related information has a memory advantage due to the self-reference effect, and this memory advantage is more obvious in the self-immersed perspective. Therefore, we observed this phenomenon only under the implicit reference condition. In addition, self-referential processing can produce a stronger DF effect, not because the self-referential information that must be forgotten is more inhibited during the retrieval process but because self-referential information that must be remembered is more easily retrieved. This finding indicates that self-referential information does not improve DF. However, individuals are able to forget self-related information, which also provides theoretical support for the psychotherapy of CSA and PTSD patients by increasing their ability to inhibit the retrieval of self-related negative emotions and traumatic experiences.

In sum, the present study showed that the item-method DF effect is the result of the selective rehearsal mechanism and the retrieval inhibition mechanism working together. Furthermore, the present study found that both self-reference and other-reference cause DF in explicit referential processing or implicit referential processing, but the DF effect is stronger under the self-referential condition. Finally, our results showed that the memory advantage effect of implicit self-referential processing is stronger than that of explicit self-referential processing.

#### Research limitations and prospects

Some limitations of our study should be mentioned. First, we used a storage-extraction model in which participants needed to learn a word pair before the instructions were presented. Under the premise of ensuring sufficient learning materials in each experimental condition, participants had to learn twice the number of materials, which may have caused the participants’ memory to be overloaded (especially in the experimental group) and may have decreased the recall rate. Explicit self-referential processing that did not appear to have a memory advantage effect could also be caused by this reason. Additionally, in the Chinese language system, it is difficult to avoid the situation in which synonyms represent the same kind of thing, which led the participants to produce a few erroneous memories after learning many materials. To ensure the objectiveness and accuracy of our experiment, we did not include false memory in the recall rate. Whether this false memory can be completely disregarded remains to be discussed. In the present study, to present a situation more similar to people’s lives, we used the item-method DF paradigm. However, the list-method DF also reflects some real-life situations. For example, students reviewed a few knowledge points before the exam, and then, the teacher told them that these knowledge points would not be tested. Therefore, they had to forget the content that they had previously reviewed and to remember other knowledge points. In future research, the list-method DF paradigm and the storage-retrieval MPT model can be used to explore the internal psychological mechanism of list-method DF combined with specific situations. In addition, both memory and forgetting involve the storage and retrieval of information, so we can apply the storage-retrieval MPT model to studies of memory or forgetting in different fields. Finally, since we did not provide manipulation checks for implicit manipulation in Experiment 2, it might be more appropriate to use "subtle" rather than "implicit". Therefore, we hope that subsequent studies will improve it.

## Supporting information

S1 Table Probability estimate for all parameters between experimental group and control group in Experiments 1 and 2. TBF/R items were items for which participants in the experimental group received “forget” cues, whereas participants in the control group received “remember” cues. For TBR/R items, all participants received “remember” cues.
(DOCX)
