# Supplementary material for: Can self-referential information improve directed forgetting? Evidence from a multinomial processing tree model
Source: PLoS One. 2019 Jan 28;14(1):e0211280. doi: 10.1371/journal.pone.0211280 (PMC6349339; doi:10.1371/journal.pone.0211280)
Supplement: S1 Table — TBF/R items were items for which participants in the experimental group received “forget” cues, whereas participants in the control group received “remember” cues. For TBR/R items, all participants received “remember” cues. (DOCX) [file pone.0211280.s001.docx]

|  |  | **Experimental Group** |  |  | **Control Group** |  |
| --- | --- | --- | --- | --- | --- | --- |
| Parameter | *M* | *SE* | *95% CI* | *M* | *SE* | *95% CI* |
|  |  |  | **Experiment 1** |  |  |  |
| ***a* parameter** |  |  |  |  |  |  |
| *Self-Reference* |  |  |  |  |  |  |
| TBF/R-items | 0.179 | 0.028 | [0.125, 0.233] | 0.424 | 0.036 | [0.354, 0.495] |
| TBR/R-items | 0.572 | 0.036 | [0.502, 0.642] | 0.440 | 0.036 | [0.368, 0.511] |
| *Other-Reference* |  |  |  |  |  |  |
| TBF/R-items | 0.213 | 0.030 | [0.156, 0.271] | 0.420 | 0.036 | [0.350, 0.489] |
| TBR/R-items | 0.522 | 0.037 | [0.450, 0.594] | 0.434 | 0.036 | [0.364, 0.504] |
| ***r* parameter** |  |  |  |  |  |  |
| *Self-Reference* |  |  |  |  |  |  |
| TBF/R-items | 0.163 | 0.062 | [0.042, 0.284] | 0.396 | 0.054 | [0.290, 0.502] |
| TBR/R-items | 0.659 | 0.046 | [0.569, 0.748] | 0.338 | 0.051 | [0.238, 0.438] |
| *Other-Reference* |  |  |  |  |  |  |
| TBF/R-items | 0.296 | 0.070 | [0.159, 0.433] | 0.430 | 0.055 | [0.324, 0.537] |
| TBR/R-items | 0.467 | 0.050 | [0.369, 0.566] | 0.434 | 0.054 | [0.328, 0.540] |
| ***s* parameter** |  |  |  |  |  |  |
| *Self-Reference* |  |  |  |  |  |  |
| TBF/R-items | 0.034 | 0.024 | [-0.013, 0.081] | 0.101 | 0.034 | [0.035, 0.167] |
| TBR/R-items | 0.138 | 0.046 | [0.049, 0.228] | 0.067 | 0.025 | [0.017, 0.116] |
| *Other-Reference* |  |  |  |  |  |  |
| TBF/R-items | 0.036 | 0.025 | [-0.014, 0.085] | 0.058 | 0.026 | [0.007, 0.108] |
| TBR/R-items | 0.116 | 0.035 | [0.048, 0.183] | 0.093 | 0.033 | [0.029, 0.157] |
| ***u* parameter** |  |  |  |  |  |  |
| *Self-Reference* |  |  |  |  |  |  |
| TBF/R-items | 0.000 | 0.055 | [-0.108, 0.108] | 0.038 | 0.013 | [0.012, 0.064] |
| TBR/R-items | 0.058 | 0.019 | [0.020, 0.096] | 0.050 | 0.016 | [0.019, 0.082] |
| *Other-Reference* |  |  |  |  |  |  |
| TBF/R-items | 0.019 | 0.008 | [0.003, 0.034] | 0.033 | 0.012 | [0.010, 0.056] |
| TBR/R-items | 0.053 | 0.018 | [0.018, 0.089] | 0.047 | 0.014 | [0.018, 0.075] |
| ***l* parameter** |  |  |  |  |  |  |
| *Self-Reference* |  |  |  |  |  |  |
| TBF/R-items | 0.053 | 0.014 | [0.026, 0.080] | – | – | – |
| TBR/R-items | – | – | – | – | – | – |
| *Other-Reference* |  |  |  |  |  |  |
| TBF/R-items | – | – | – | – | – | – |
| TBR/R-items | – | – | – | – | – | – |

|  |  | **Experimental Group** |  |  | **Control Group** |  |
| --- | --- | --- | --- | --- | --- | --- |
| Parameter | *M* | *SE* | *95% CI* | *M* | *SE* | *95% CI* |
|  |  |  | **Experiment 2** |  |  |  |
| ***a* parameter** |  |  |  |  |  |  |
| *Self-Reference* |  |  |  |  |  |  |
| TBF/R-items | 0.275 | 0.034 | [0.208, 0.342] | 0.516 | 0.038 | [0.441, 0.591] |
| TBR/R-items | 0.628 | 0.037 | [0.555, 0.700] | 0.550 | 0.038 | [0.475, 0.625] |
| *Other-Reference* |  |  |  |  |  |  |
| TBF/R-items | 0.235 | 0.032 | [0.171, 0.298] | 0.506 | 0.038 | [0.431, 0.581] |
| TBR/R-items | 0.569 | 0.038 | [0.494, 0.644] | 0.527 | 0.039 | [0.451, 0.603] |
| ***r* parameter** |  |  |  |  |  |  |
| *Self-Reference* |  |  |  |  |  |  |
| TBF/R-items | 0.182 | 0.055 | [0.074, 0.289] | 0.451 | 0.052 | [0.348, 0.553] |
| TBR/R-items | 0.676 | 0.045 | [0.587, 0.764] | 0.432 | 0.050 | [0.334, 0.531] |
| *Other-Reference* |  |  |  |  |  |  |
| TBF/R-items | 0.213 | 0.063 | [0.089, 0.337] | 0.383 | 0.051 | [0.282, 0.484] |
| TBR/R-items | 0.490 | 0.050 | [0.392, 0.589] | 0.340 | 0.050 | [0.243, 0.437] |
| ***s* parameter** |  |  |  |  |  |  |
| *Self-Reference* |  |  |  |  |  |  |
| TBF/R-items | 0.003 | 0.003 | [-0.003, 0.009] | 0.044 | 0.013 | [0.020, 0.069] |
| TBR/R-items | 0.084 | 0.021 | [0.043, 0.124] | 0.034 | 0.011 | [0.012, 0.056] |
| *Other-Reference* |  |  |  |  |  |  |
| TBF/R-items | 0.021 | 0.008 | [0.005, 0.036] | 0.028 | 0.010 | [0.009, 0.048] |
| TBR/R-items | 0.064 | 0.017 | [0.031, 0.096] | 0.056 | 0.014 | [0.027, 0.084] |
| ***u* parameter** |  |  |  |  |  |  |
| *Self-Reference* |  |  |  |  |  |  |
| TBF/R-items | 0.003 | 0.003 | [-0.003, 0.009] | 0.044 | 0.013 | [0.020, 0.069] |
| TBR/R-items | 0.084 | 0.021 | [0.043, 0.124] | 0.034 | 0.011 | [0.012, 0.056] |
| *Other-Reference* |  |  |  |  |  |  |
| TBF/R-items | 0.021 | 0.008 | [0.005, 0.036] | 0.028 | 0.010 | [0.009, 0.048] |
| TBR/R-items | 0.064 | 0.017 | [0.031, 0.096] | 0.056 | 0.014 | [0.027, 0.084] |
| ***l* parameter** |  |  |  |  |  |  |
| *Self-Reference* |  |  |  |  |  |  |
| TBF/R-items | 0.038 | 0.012 | [0.015, 0.062] | – | – | – |
| TBR/R-items | – | – | – | – | – | – |
| *Other-Reference* |  |  |  |  |  |  |
| TBF/R-items | – | – | – | – | – | – |
| TBR/R-items | – | – | – | – | – | – |
